# DBC1 maintains skeletal muscle integrity by enhancing myogenesis and preventing myofibre wasting

**DOI:** 10.1002/jcsm.13398

**Published:** 2023-12-07

**Authors:** Na Liang, Jia He, Jiaqi Yan, Xueying Han, Xiaoqian Zhang, Yamei Niu, Wuga Sha, Jun Li

**Affiliations:** ^1^ State Key Laboratory of Common Mechanism Research for Major Diseases, Institute of Basic Medical Sciences Chinese Academy of Medical Sciences and Peking Union Medical College Beijing China; ^2^ Department of Pathology, Institute of Basic Medical Sciences Chinese Academy of Medical Sciences Beijing China; ^3^ School of Basic Medicine Peking Union Medical College Beijing China; ^4^ Neuroscience Center Chinese Academy of Medical Sciences Beijing China; ^5^ Molecular Pathology Research Center Chinese Academy of Medical Sciences and Peking Union Medical College Beijing China

**Keywords:** DBC1, FOXO3, myogenesis, skeletal muscle atrophy

## Abstract

**Background:**

Skeletal muscle atrophy, particularly ageing‐related muscular atrophy such as sarcopenia, is a significant health concern. Despite its prevalence, the underlying mechanisms remain poorly understood, and specific approved medications are currently unavailable. Deleted in breast cancer 1 (DBC1) is a well‐known regulator of senescence, metabolism or apoptosis. Recent reports suggest that DBC1 may also potentially regulate muscle function, as mice lacking DBC1 exhibit weakness and limpness. However, the function of DBC1 in skeletal muscle and its associated molecular mechanisms remain unknown, thus prompting the focus of this study.

**Methods:**

Tibialis anterior (TA) muscle‐specific DBC1 knockdown C57BL/6J male mice were generated through a single injection of 2.00 E + 11 vg of adeno‐associated virus 9 delivering single‐guide RNA for DBC1. Grip strength and endurance were assessed 2 months later, followed by skeletal muscle harvest. Muscle atrophy model was generated by cast immobilization of the mouse hindlimb for 2 weeks. Molecular markers of atrophy were probed in muscles upon termination. Cardiotoxin (CTX) was injected in TA muscles of DBC1 knockdown mice, and muscle regeneration was assessed by immunohistochemistry, quantitative PCR and western blotting. DBC1 knockdown C2C12 cells and myotubes were investigated using immunofluorescence staining, Seahorse, immunohistology, fluorescence‐activated cell sorting and RNA‐sequencing analyses.

**Results:**

DBC1 knockdown in skeletal muscle of young mice led to signatures of muscle atrophy, including a 28% reduction in muscle grip force (*P* = 0.023), a 54.4% reduction in running distance (*P* = 0.002), a 14.3% reduction in muscle mass (*P* = 0.007) and significantly smaller myofibre cross‐sectional areas (*P* < 0.0001). DBC1 levels decrease in age‐related or limb immobilization‐induced atrophic mouse muscles and overexpress DBC1‐attenuated atrophic phenotypes in these mice. Muscle regeneration was hampered in mice with CTX‐induced muscle injury by DBC1 knockdown, as evidenced by reductions in myofibre cross‐sectional areas of regenerating myofibres with centralized nuclei (*P* < 0.0001), percentages of MyoG^+^ nuclei (*P* < 0.0001) and fusion index (*P* < 0.0001). DBC1 transcriptionally regulated mouse double minute 2 (MDM2), which mediated ubiquitination and degradation of forkhead box O3 (FOXO3). Increased FOXO3 proteins hampered myogenesis in DBC1 knockdown satellite cells by compromising around 50% of mitochondrial functions (*P* < 0.001) and exacerbated atrophy in DBC1 knockdown myofibres by activating the ubiquitin–proteasome and autophagy–lysosome pathways.

**Conclusions:**

DBC1 is essential in maintaining skeletal muscle integrity by protecting against myofibres wasting and enhancing muscle regeneration via FOXO3. This research highlights the significance of DBC1 for healthy skeletal muscle function and its connection to muscular atrophy.

## Introduction

Skeletal muscle atrophy, characterized by decreased muscle mass and function, threatens the quality of life.^1^ Muscle atrophy can occur as a result of physiological or pathological factors, such as ageing,^2^ ^,^ ^S1^ immobilization^3^ or diseases.^4^ ^,^ ^S2^ Skeletal muscle is a heterogeneous tissue that consists of mononuclear muscle stem cells (satellite cells) and multinuclear myofibres.^5^ Satellite cells, the multipotent stem cells that are responsible for skeletal muscle regeneration,^6^ ^,^ ^S3^ activate and finally form terminally differentiated myofibres upon skeletal muscle injury to maintain muscle integrity.^7^ Myofibre is the main cell type of skeletal muscle, and its size and quality determine muscle mass and function.^8^ Weakened satellite cells' myogenic capacity and accelerated myofibre wasting are significant contributors of muscle atrophy.^9^ ^,^ ^S4,S5^ Mitochondrial dysfunctions are important factors for impaired myogenic capacity of satellite cells,^10^ ^,^ ^S6^ and excessive degradation of myofibrillar proteins, controlled by abnormal activation of the ubiquitin–proteasome and autophagy–lysosome pathways,^11^ ^,^ ^S7^ is an important cause or feature of myofibre wasting.^12^ Despite significant advances in understanding the mechanisms that regulate muscles loss in diseases, effective pharmacological treatments for atrophying muscle are currently unavailable, necessitating a new perspective of mechanistic insight. Recent studies reveal that common coordinators exist, which regulate functions of muscle cells at different developmental stages. For example, Ca^2+^ acts as a coordinator in both satellite cell myogenic differentiation and myotube maturation,^13^ and palladin, a scaffolding microfilament‐associated phosphoprotein, is reported to inhibit early myogenesis but promotes myotube maturation.^14^ Discovering these coordinators might open a new avenue of combating muscle atrophy.

Deleted in breast cancer 1 (DBC1) is a large multidomain protein that regulates various cellular functions, including apoptosis, metabolism, tumourigenesis and DNA repair.^15^ ^,^ ^S8^ Emerging evidences suggest that DBC1 may also play a role in regulating muscle functions. Studies have shown that DBC1 deletion mice are more susceptible to aortic dissections upon angiotensin II (ANG II), partially due to reduced activation of vascular smooth muscle cells in the arterial walls.^16^ Additionally, DBC1 knockout mice also exhibit symptoms of experimental autoimmune myasthenia gravis (EAMG), such as severe limb weakness, presumably due to the inhibition of NF‐κB pathway.^17^ Moreover, DBC1 has been shown to inhibit sirtuin 1 (SIRT1) activity,^18^ which has been linked to negative regulation of satellite cell differentiation.^19^ ^,^ ^S9^ These findings suggest that DBC1 plays an important role in regulating muscle functions that have yet to be fully elucidated. Further research is necessary to clarify the specific roles of DBC1 in skeletal muscle.

Forkhead box O3 (FOXO3) is the main member of forkhead box O (FOXO) family that is expressed in muscles and plays a key regulator of atrophy.^20^ FOXO3 induces the expression of atrophy‐related ubiquitin ligases Atrogin1 and Murf1, which are the main factors of ubiquitin–proteasome pathway responsible for protein degradation and muscle loss.^11^ ^,^ ^S10^ Additionally, FOXO3 activates the autophagy–lysosome pathway by inducing expression of autophagy‐related genes, such as light chain 3 (LC3).^21^ FOXO3 is also highly expressed in quiescent satellite cells and maintains their quiescence,^22^ ^,^ ^S11^ and its absence hampers self‐renewal of satellite cells, leading to myogenesis.^23^ ^,^ ^S12^ However, the precise mechanism by which FOXO3 regulates myogenesis remains unclear.

In this study, we hypothesize that DBC1 plays a regulatory role in maintaining skeletal muscle integrity. Our findings reveal that DBC1 plays an essential role in enhancing myogenesis and repressing myofibre wasting. Specifically, we demonstrated that DBC1 negatively regulated FOXO3 protein homeostasis through mouse double minute 2 (MDM2)‐mediated ubiquitination system. As a result, DBC1 deficiency led to increased FOXO3 protein levels, which impaired myogenic capacity of satellite cells by disrupting mitochondrial functions and led to myofibre wasting via activating ubiquitin–proteasome and autophagy–lysosome pathways. Our study suggests that DBC1 regulates myogenesis and myofibre maintenance via the MDM2/FOXO3 pathway, shedding light on the mechanisms underlying age‐related muscle atrophy and offering potential new perspectives for combating skeletal muscle atrophy.

## Methods

### Cell culture

C2C12 cells (BMCR) were cultured in Dulbecco's modified Eagle's medium (Corning, 10‐013‐CVRC) containing 10% foetal bovine serum (FBS) (Gibco, 16140071) and 1% penicillin/streptomycin (Solarbio, P1400). For differentiation of C2C12 myoblasts into myotubes, cells were transferred to DMEM containing 2% horse serum (Thermo, 26050070) and then changing fresh medium every 2 days.

### Mouse handling

All C57BL/6 mice were purchased from SPF Biotechnology (Beijing, China). Mice were housed in specific pathogen‐free (SPF) barrier facilities. All procedures were approved by the Animal Ethics Committee of Peking Union Medical College. DBC1 knockdown mice were performed by intramuscular injection of 2.00 E + 11 vg of adeno‐associated virus 9 (AAV9) (BrainVTA, Wuhan China) carrying single‐guide RNA (sgRNA) against DBC1 or a scram‐bled sgRNA control into hindlimb tibialis anterior (TA) muscles.

### Grip force test

The muscle forces of DBC1 knockdown and control mice were evaluated by using a BIO‐GS3 claw grip tester. Each mouse was tested for five times, and the data collected are the average of the three highest values out of the five tests.

### Endurance test

Muscle endurance capacity was evaluated by using a flat motorized treadmill (ZH‐PT/5S, Zhenghua Biology). The test started at a speed of 10 m/min, followed by 12, 15 and 18 m/min for 5 min and maintained at 20 m/min till the endpoint. The experiment ended when the mice stopped running and did not attempt to run again after being electrocuted more than 10 times.

### Immunoblotting

Samples were homogenized in lysis buffer (50‐mM tris, pH 7.5, 150‐mM NaCl, 0.5% NP‐40) supplemented withprotease inhibitor cocktail (MedChemExpress [MCE], HY‐K0010), followed by SDS‐PAGE and electrotransferred to 0.45‐μm polyvinylidene difluoride (PVDF) membrane (Millipore). Membranes were blocked with 5% non‐fat dry milk in tris‐buffered saline with Tween 20 (TBST) for 1 h and then incubated with primary antibodies overnight at 4°C, followed by incubation with proper secondary antibodies. Antibodies were used at the following concentrations: anti‐DBC1 (1:2000, Bethyl, A300434A), anti‐myogenin (MyoG) (1:500, Santa Cruz, sc‐52903), anti‐myosin heavy chain (MHC) (1:1000, Developmental Studies Hybridoma Bank [DSHB], MF20‐C), anti‐FOXO3 (1:2000, Cell Signaling Technology [CST], 2497S), anti‐Atrogin1 (1:500, Santa Cruz, sc‐16806), anti‐Murf1 (1:500, Santa Cruz, sc‐398608), anti‐LC3 (1:500, ABclonal, A19665), anti‐MDM2 (1:500, Santa Cruz, sc‐13161), anti‐ubiquitin (1:500, Santa Cruz, sc‐8017), anti‐β‐tubulin (1:5000, CMCTAG, AT0003) and anti‐GAPDH (1:5000, Millipore, mab374). Blotting signals were visualized on Azure 200 Gel Imager (Azure Biosystems).

### Immunofluorescence

Muscle sections (10 μm) were fixed with 4% polyformaldehyde for 30 min, followed by three times of washing in phosphate‐buffered saline (PBS). The samples were permeabilized using PBS containing 0.5% Triton X‐100 for 15 min and then blocked with 1% bovine serum albumin for 2 h. The samples were incubated with anti‐MyoG (1:100, Santa Cruz, sc‐52903), MHC (1:100, DSHB, MF20‐C), Ki‐67 (1:500, Abcam, ab15580), anti‐DBC1 (1:200, Absin, abs117680) or anti‐FOXO3 (1:200, Absin, abs115009) overnight at 4°C. The samples were washed with PBS containing 0.05% Tween 20 for three times and then incubated with the secondary antibodies (Alexa Fluor® 568 and 488 antibodies, 1:500, Abcam) for 50 min in the dark. Nuclei were counterstained with 4′,6‐diamidino‐2‐phenylindole (DAPI), and images were taken using Lecia DM6 B Upright Microscope.

### Mitochondrial membrane potential assay

Tetramethylrhodamine methyl ester perchlorate (TMRM, Sigma, T5428) was diluted with serum‐free medium at the final concentration of 20 nM. The medium was removed and replaced with 500‐μL diluted TMRM, using 10‐μM carbonyl cyanide 4‐(trifluoromethoxy)phenylhydrazone (FCCP, MCE, 370‐86‐5) as positive control, and then cells were incubated at 37°C for 20 min. After incubation, cells were washed three times and resuspended in PBS. Fluorescein isothiocyanate (FITC) channel was selected for flow cytometry detection.

### Reactive oxygen species measurement

Dichlorodihydrofluorescein diacetate (DCFH‐DA) (Beyotime, S0033S) was diluted with serum‐free medium at the final concentration of 10 μM. The medium was removed and replaced with 500‐μL diluted DCFH‐DA, using 10‐μM rotenone (MCE, HY‐B1756) as positive control, and then cells were incubated at 37°C for 20 min. After incubation, cells were washed three times and resuspended in PBS. FITC channel was selected for flow cytometry detection.

### Mitochondria number

MitoTracker Red CMXRos (Beyotime, C1049B) was diluted with serum‐free medium at the final concentration of 100 nM. The medium was removed and replaced with 500‐μL diluted MitoTracker Red CMXRos, and then cells were incubated at 37°C for 20 min. After incubation, cells were washed three times and resuspended in PBS. FITC channel was selected for flow cytometry detection.

### Succinate dehydrogenase and glycerophosphate dehydrogenase staining

For succinate dehydrogenase (SDH) staining, the myotubes or muscle sections (10 μm) were incubated in a solution consisting of 1‐mM sodium azide, 1‐mM l‐methoxyphenazine methosulfate (MPMS, Macklin, M860955), 1.5‐mM nitroblue tetrazolium (NBT) (Macklin, N814596), 5‐mM EDTA and 100‐mM sodium phosphate buffer (pH 7.6) in the dark at 37°C for 10 min, followed by incubation in a substrate solution consisting of 48‐mM succinic acid (Macklin, L897377) for 5–10 min. For glycerophosphate dehydrogenase (GPDH) staining, the myotubes or muscle sections (14 μm) were incubated with a solution consisting of 1‐mM sodium azide, 1‐mM MPMS, 1.2‐mM NBT and 100‐mM sodium phosphate buffer (pH 7.4) at 37°C for 20 min, followed by another incubation with a substrate solution consisting of 9.3‐mM α‐glycerophosphate (Sigma, G9422) for 10–20 min. The images were captured using Lecia DM6 B Upright Microscope.

### Haematoxylin and eosin staining

Frozen tissue blocks used for haematoxylin and eosin (H&E) staining were sectioned at 10 μm. H&E staining was conducted by Wuhan Servicebio Technology Company following standard protocol. Images were taken by Leica DM6 B Upright Microscope.

### Mitochondrial bioenergetic measurements

The cells were plated in an XF 24‐well microplate (Seahorse Bioscience) at a density of 20 000 cells per well and differentiated for 2 days. Oxygen consumption rate was measured in cells at 37°C using an XF24 analyser (Seahorse Bioscience) according to manufacturer's instructions; 10‐μM oligomycin (MCE, 1404‐19‐9), 40‐μM FCCP and 10‐μM rotenone (MCE, HY‐B1756)/antimycin (Sigma, A8674) were used to detect the uncoupled respiration, maximal respiration and nonmitochondrial respiration, respectively.

### Statistical analysis

Data were expressed as mean ± standard deviation. *P* values were calculated in GraphPad Prism using two‐tailed Student's *t* test or one‐way analysis of variance (ANOVA) for multiple comparison. *P* values are denoted in figures as follows: ns (not significant), *P < 0.05, ^**^ *P* < 0.01, ^***^ *P* < 0.001 and ^****^ *P* < 0.0001.

## Results

### The deficiency of DBC1 is associated with muscle atrophy and impaired muscle regeneration

**Figure 1:**
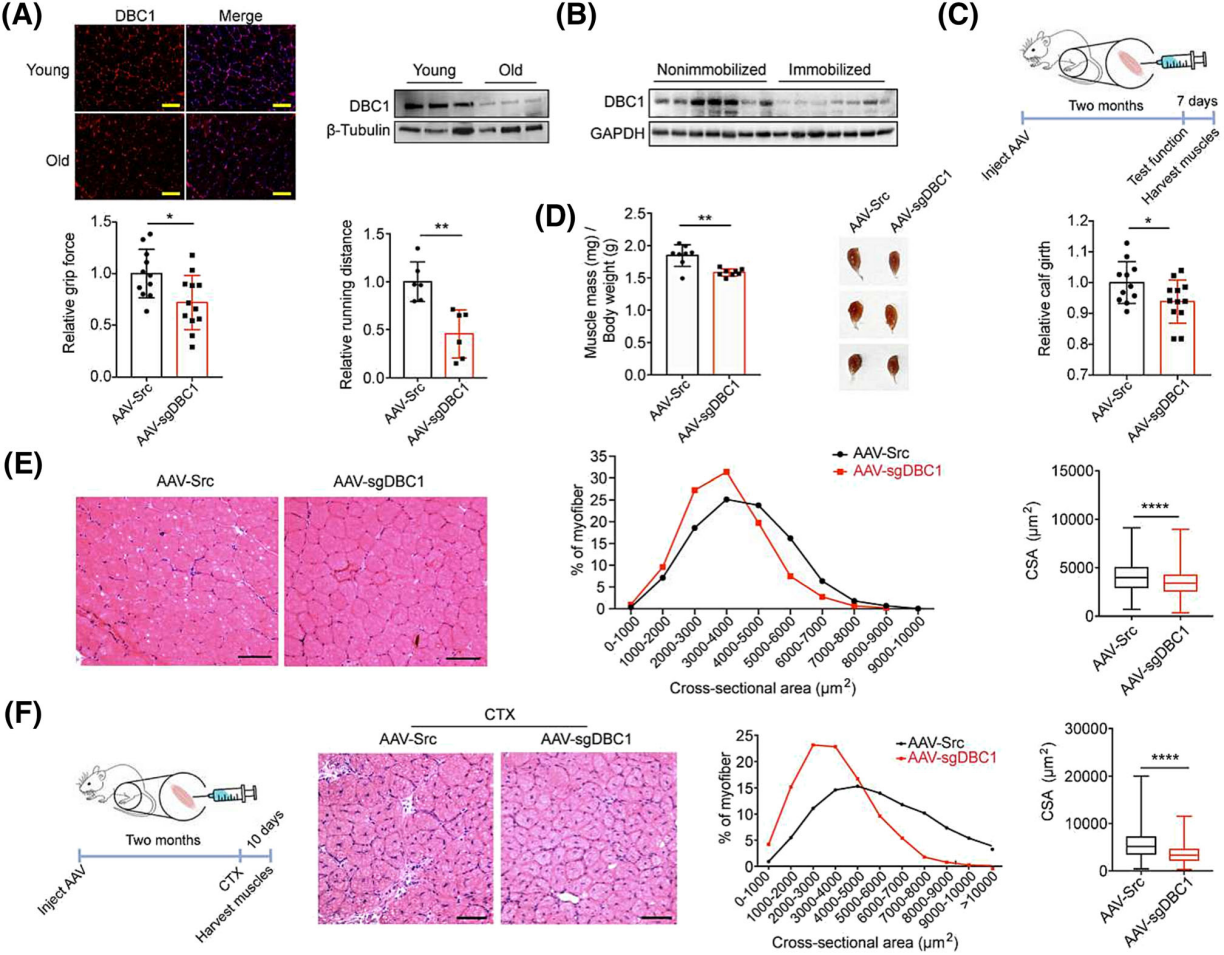
DBC1 deletion impairs skeletal muscle integrity and regeneration. (A) Immunofluorescence staining (left) and western blotting analysis (right) for DBC1 protein levels in TA muscles isolated from young (3 months) and old (24 months) mice. Nuclei were counterstained with DAPI (blue). Scale bars = 100 μm. (B) Western blotting analysis for DBC1 protein levels in TA muscles isolated from C57BL/6J mice with limbs immobilized for 3 weeks. (C) Schematic to illustrate experimental design of knocking down DBC1 in mouse TA muscles: TA muscles of 8‐week‐old male C57BL/6J mice were injected intramuscularly with AAV‐sgDBC1 or AAV‐src. Relative grip force and relative running distance (relative to those of AAV‐src control group) were tested 2 months after the injection, followed by collecting muscle samples 7 days later (*n* = 12 for each group). (D) TA muscles weight, normalized to body weight (*n* = 8 for each group) (left), representative images of TA muscles (middle) and relative calf girth (*n* = 12 for each group) (right) of DBC1 knockdown and control mice. (E) (left) Representative images of haematoxylin and eosin (H&E) staining of TA muscles isolated from DBC1 knockdown and control mice. Scale bar = 100 μm. (right) Percentage distribution of myofibre cross‐sectional area (CSA) (middle) and the mean CSA (right) in TA muscles of DBC1 knockdown and control mice calculated from the H&E staining sections was quantified. Data were pooled from 2502 myofibres for control mice and 3285 myofibres for DBC1 knockdown mice (four mice for each group). (F) (left) Schematic to illustrate regeneration of TA muscles in DBC1 knockdown mice: TA muscles from 8‐week‐old C57BL/6J mice were injected intramuscularly with AAV‐sgDBC1 or AAV‐src for 2 months to knock down DBC1 and then were damaged by injecting 50‐μL cardiotoxin (CTX, 10 μM), intramuscularly. Muscle samples were collected 10 days after CTX injection. (right) Representative images of H&E staining of TA muscles isolated from DBC1 knockdown or control mice 10 days after CTX‐induced damage. Percentage distribution of CSA and mean CSA of regenerating myofibres with central nuclei, calculated from the H&E staining sections, was quantified. Data were pooled from 4392 myofibres for control mice and 5850 myofibres for DBC1 knockdown mice (four mice for each group). All *P* values were calculated using two‐tailed Student's *t* test.

In atrophic skeletal muscle of old mice (*Figure* *1* *A*), mice with limb immobilization (*Figure* *1* *B*) and cachexia mice (*Figure* *S1* *a*–*c*), a significant reduction in DBC1 protein levels was observed, suggesting a potential role of DBC1 in muscle atrophy. To investigate this further, we created TA muscle‐specific DBC1 knockdown mice by intramuscularly injecting adeno‐associated virus (AAV9) particles delivering sgRNA for DBC1 or scrambled control into TA muscles of 3‐month‐old C57BL/6 mice (*Figure* *1* *C*). Two months after virus injection, DBC1 was significantly reduced (*Figure* *S1* *d*), whereas DBC1 knockdown had no effect on body weight of mice (*Figure* *S1* *e*). In DBC1 knockdown mice, the grip force and endurance of muscles were significantly decreased (*Figure* *1* *C*); the loss of muscle mass was greater as also evidenced by smaller calf girth (*Figure* *1* *D*). Consistently, after DBC1 knockdown in TA muscles, the myofibre cross‐sectional areas (CSAs) were also significantly reduced (*Figure* *1* *E*), suggesting shrinkage of myofibres. These data indicate that lacking DBC1 in TA muscles causes muscle atrophy. Considering that dysfunctional myogenetic capacity of satellite cells also contributes to muscle atrophy,^24^ we wondered whether DBC1 knockdown affects myogenesis. We introduced cardiotoxin (CTX)‐mediated injury in TA muscles of control and DBC1 knockdown mice (*Figure* *1* *F*). Ten days after injury, TA muscle mass remained remarkedly reduced in DBC1 knockdown mice (*Figure* *S2* *a*–*c*). The CSAs of myofibres with centralized nuclei were significantly decreased, where the fluorescence signals of MyoD or MyoG were detected (*Figures* *1* *F* and *S2* *d*,*e*). At all, these results indicate that DBC1 knockdown results in muscle atrophy and hinders muscle regeneration.

### DBC1 modulates myogenesis

**Figure 2:**
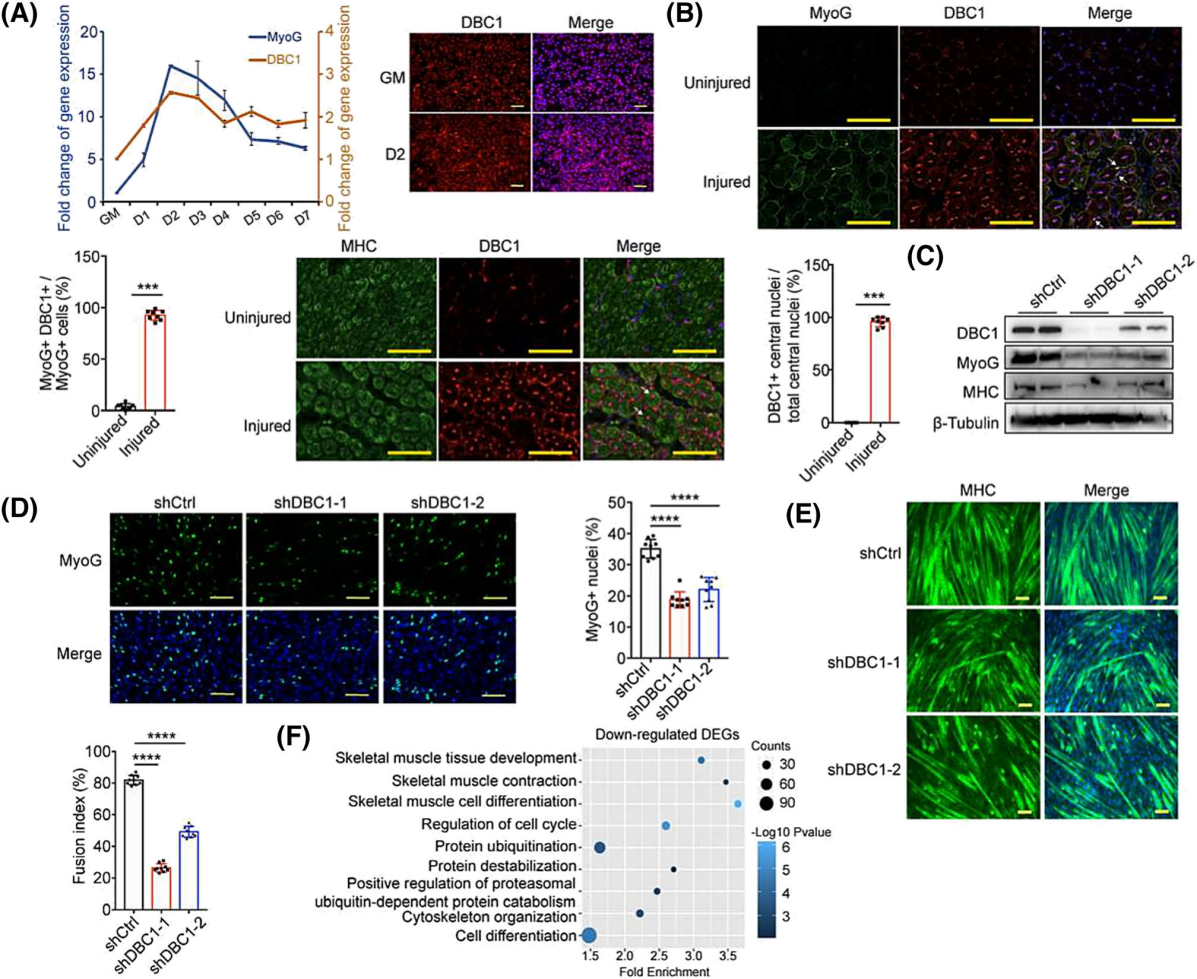
DBC1 regulates myogenesis. (A) (left) Time‐course gene expression of *DBC1* and *MyoG* in proliferating and differentiating C2C12 cells, determined by RT‐qPCR. GM represents proliferating stage. D1, D2, D3, D4, D5, D6 and D7 represent a differentiating time of 1, 2, 3, 4, 5, 6 or 7 days, respectively. (right) Immunofluorescence staining of DBC1 (red) in proliferating C2C12 cells (GM) and C2C12 cells that had been induced to differentiate for 2 days (D2). Nuclei were counterstained with DAPI (blue). Scale bars = 100 μm. (B) Immunofluorescence staining of MyoG (green) and DBC1 (red) as well as MHC (green) and DBC1 (red) in TA muscles that were damaged by CTX for 5 days (injured) or not (uninjured). Nuclei were counterstained with DAPI (blue). Arrowheads indicate representative cells express DBC1 and MyoG meanwhile or central nuclei express DBC1. Scale bars = 100 μm. The percentages of DBC1‐expressing MyoG^+^ myoblasts measured by the ratio of MyoG^+^/DBC1^+^ cells compared to MyoG^+^ myoblasts. Percentages of DBC1‐expressing central nuclei measured by the ratio of DBC1^+^ central nuclei compared to total central nuclei. (C) Western blotting analysis for DBC1, MyoG and MHC protein levels in DBC1 knockdown and control C2C12 cells that had been induced to differentiate for 2 days. (D) (left) Immunofluorescence staining of MyoG (green) in DBC1 knockdown and control C2C12 cells that had been induced to differentiate for 2 days. Nuclei were counterstained with DAPI (blue). Scale bars = 100 μm. (right) The proportion of MyoG^+^ nuclei was quantified. (E) Immunofluorescence staining of MHC (green) and quantification of the fusion index in DBC1 knockdown and control C2C12 cells that had been induced to differentiate for 7 days. Nuclei were counterstained with DAPI (blue). Scale bars = 100 μm. (F) Gene Ontology (GO) analysis of the downregulated genes in DBC1 knockdown C2C12 cells, performed with −log_10_ (*P* value) plotted as a function of classification meeting a *P* value of <0.05. *P* values were calculated using two‐tailed Student's *t* test (B) or one‐way ANOVA for multiple comparison (D, E).

To further investigate the involvement of DBC1 in myogenesis, we utilized C2C12 cells as an in vitro myoblast model. *DBC1* mRNA expression was found to increase during C2C12 cell differentiation in a similar pattern to *MyoG*, the early marker of myogenesis (*Figure* *2* *A*). The increased expression of DBC1 during myogenesis was confirmed by immunofluorescence staining, which revealed a greater abundance of DBC1 proteins on the second day of differentiation (D2) (*Figure* *2* *A*). To verify this in vivo, we injured TA muscles of the wild‐type mice with intramuscular CTX injection (*Figure* *S3* *a*) and co‐stained DBC1 with markers for different types of muscle cells. The results showed that DBC1 protein levels were significantly increased 5 days after injury in 92.5% of MyoG^+^ cells, which represent stem cell‐derived myoblasts, and 95.6% of central nuclei of MHC^+^ cells, which represent newly formed myofibres (*Figure* *2* *B*), but not in the satellite cells, as indicated by the unchanged ratio of DBC1^+^/Pax7^+^ cells and Pax7^+^ cells (*Figure* *S3* *b*). These results imply that the increase of DBC1 proteins occurs only during myogenesis. Consistently, knockdown of DBC1 in C2C12 cells led to a significant inhibition of myogenesis, as evidenced by reduced MyoG and MHC protein levels (*Figure* *2* *C*) and decreased percentages of MyoG^+^ nuclei (*Figure* *2* *D*), as well as reduced fusion index (*Figure* *2* *E*). RNA‐sequencing analysis of DBC1 knockdown C2C12 cells further confirmed the necessity of DBC1 in myogenesis. We identified 3936 genes whose expression was significantly modulated after DBC1 knockdown, of which 2101 were downregulated (*Figure* *S4*). Gene Ontology (GO) analysis of these downregulated genes revealed enrichment in skeletal muscle cell differentiation and skeletal muscle development pathways (*Figure* *2* *F*), which further demonstrates the importance of DBC1 for myogenesis. DBC1 was reported to affect cell cycle and apoptosis.^15^ Notably, we ruled out the possibility that the impaired myogenesis in DBC1 knockdown cells was due to changes in cell proliferation or apoptosis, as there were no changes in proliferation rate or percentages of apoptotic cells observed (*Figure* *S5*). Overall, these results provide compelling evidence that DBC1 plays an indispensable role in myogenesis.

### DBC1 knockdown results in myotube wasting

**Figure 3:**
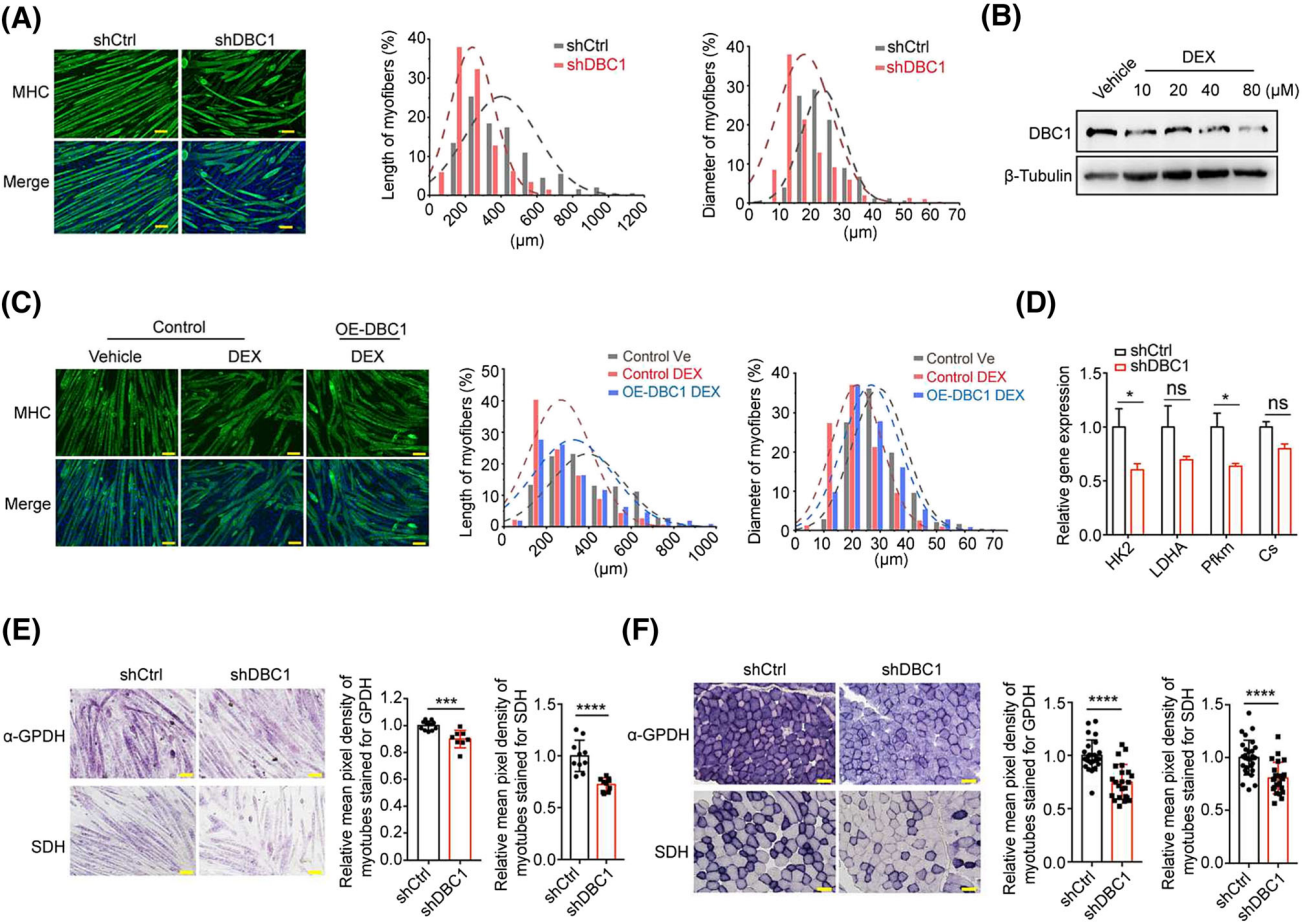
Lacking DBC1 accelerates myofibre loss. (A) (left) Immunofluorescence staining of MHC (green) in myotubes with DBC1 knockdown. (right) Percentage distribution of length and diameter of myotubes was quantified. C2C12 cells were induced to fully differentiate for 7 days and then were added with lentivirus to knock down DBC1 for 2 days, and then MHC was immunostained after knocking down DBC1, with nuclei counterstained by DAPI (blue). Scale bar = 100 μm. (B) Western blotting analysis for DBC1 protein levels in myotubes (fully differentiated for 7 days) that were treated with dexamethasone (DEX) at the dose of 10, 20, 40 or 80 μM, respectively, for 24 h. (C) (left) Immunofluorescence staining of MHC (green) in control myotubes, DEX‐treated control myotubes and DEX‐treated DBC1‐overexpressing myotubes, with nuclei counterstained by DAPI (blue). (right) Percentage distribution of the length and diameter of myotubes. C2C12 cells were induced to fully differentiate for 5 days before being infected with the control retrovirus or the retrovirus overexpressing DBC1 for 48 h. After DBC1 was overexpressed, DEX (80 μM) was added for 24 h. Scale bar = 100 μm. (D) Relative gene expression of *HK2*, *LDHA*, *Pfkm* and *Cs* in DBC1 knockdown or control myotubes; the procedure of treating myotubes was same as described in (A). (E) (left) Representative histochemical staining for α‐GPDH and SDH enzymatic activities in DBC1 knockdown myotubes. (right) Quantification of mean pixel density of myotubes stained for α‐GPDH and SDH. (F) (left) Representative histochemical staining for α‐GPDH and SDH enzymatic activities in the TA muscles of DBC1 knockdown mice. (right) Quantification of mean pixel density of myofibres stained for α‐GPDH and SDH. All *P* values were calculated using two‐tailed Student's *t* test.

Myofibre shrinkage, which is a characteristic of muscle atrophy,^25^ was observed in skeletal muscle of DBC1 knockdown mice (*Figure* *1* *E*), raising the possibility that DBC1 may affect myofibre wasting. To investigate this, we again knocked down DBC1 in fully differentiated myotubes (*Figure* *S6* *a*) and observed similar results to those in vivo, with the myotubes becoming shorter in length and thinner in diameter (*Figure* *3* *A*). Dexamethasone (DEX) and serum fast are known to induce muscle atrophy,^26^ and in these cases, DBC1 protein level decreased (*Figures* *3* *B* and *S6* *b*), while overexpression of DBC1 improved the DEX and serum fast induced myotube wasting to some extent (*Figures* *3* *C* and *S6* *c*,*d*). Consistently, DBC1 overexpression in old mice and mice with limb immobilization also significantly suppressed muscle atrophy (*Figure* *S6* *e*,*f*). Along with atrophy, the metabolism of myofibres declined.^27^ DBC1 knockdown led to significant reductions in the expression of metabolic genes, especially those related to glycolysis such as *HK2* and *Pfkm* (*Figure* *3* *D*). Histochemical staining showed that enzymatic activities of α‐GPDH and SDH decreased in DBC1 knockdown myotubes as well (*Figure* *3* *E*). Similar decreases were observed in the TA muscles with DBC1 deletion (*Figure* *3* *F*). Collectively, these findings suggest that DBC1 is likely involved in suppressing myofibre wasting.

### DBC1 regulates myogenesis and myofibre homeostasis by suppressing FOXO3

To understand how DBC1 regulates myogenesis and myofibre homeostasis, we investigated the potential involvement of SIRT1, as DBC1 inhibits SIRT1 activity,^28^ which inhibits myogenesis.^19^ However, treatment with SIRT1 inhibitor Ex‐527 (*Figure* *S7* *a*,*b*) or knockdown of SIRT1 (*Figure* *S7* *c*–*e*) in DBC1 knockdown C2C12 cells failed to rescue the bad differentiation, as confirmed by non‐significant increase of the percentages of MyoG^+^ nuclei and the fusion index, indicating that DBC1's effect on myogenesis was not dependent on SIRT1.

**Figure 4:**
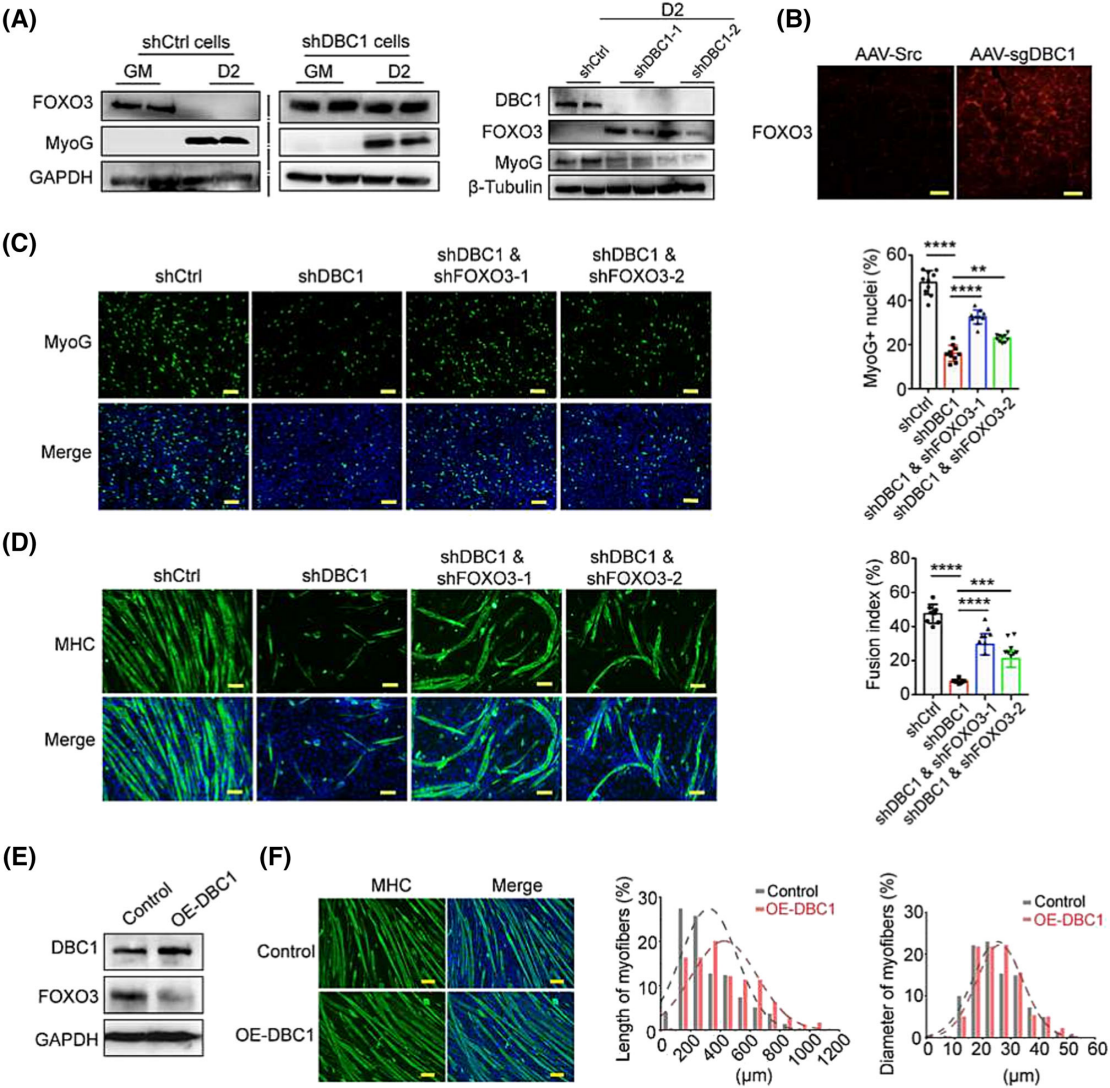
DBC1 governs myogenesis and myotube maintenance by suppressing FOXO3 protein levels. (A) (left) Western blotting analysis for FOXO3 and MyoG protein levels in proliferating (GM) and differentiating (differentiate for 2 days [D2]) control C2C12 cells or DBC1 knockdown C2C12 cells. (right) Western blotting analysis for FOXO3 and MyoG protein levels in DBC1 knockdown or control C2C12 cells that had been induced to differentiate for 2 days (D2). (B) Immunofluorescence staining of FOXO3 (red) in DBC1 knockdown TA muscles of mice that were damaged by CTX, as described in *Figure* *1*. Nuclei were counterstained with DAPI (blue). Scale bars = 100 μm. (C) (left) Immunofluorescence staining of MyoG (green) in DBC1 knockdown, DBC1 and FOXO3 double knockdown or control C2C12 cells that had been induced to differentiate for 2 days. Nuclei were counterstained with DAPI (blue). Scale bars = 100 μm. (right) Quantification of the proportion of MyoG^+^ nuclei. (D) (left) Immunofluorescence staining of MHC (green) in DBC1 knockdown, DBC1 and FOXO3 double knockdown or control C2C12 cells that had been induced to differentiate for 7 days. Nuclei were counterstained with DAPI (blue). Scale bars = 100 μm. (right) Quantification of the fusion index. (E) Western blotting analysis for FOXO3 protein levels in myotubes after DBC1 overexpression. Before subjected to western blotting, the myotubes had been induced to differentiate for 7 days followed by infection of retrovirus to overexpress DBC1 for 2 days. (F) (left) Immunofluorescence staining of MHC (green) in DBC1 overexpression myotubes and control myotubes as described in (E). Nuclei were counterstained with DAPI (blue). Scale bar = 100 μm. (right) Quantification of myotube length and diameter. *P* values were calculated using one‐way ANOVA for multiple comparison (C, D).

FOXO3, whose expression remarkably decreased upon satellite cells entering myogenic process, was reported to inhibit myogenesis.^22^ In differentiating C2C12 cells, we observed a dramatic decrease in FOXO3 protein levels compared to the proliferating cells, which disappeared in DBC1 knockdown cells (*Figure* *4* *A*). Knocking down DBC1 increased FOXO3 levels during myogenesis (*Figure* *4* *A*), and overexpressing DBC1 reversed the increase (*Figure* *S8* *a*). Consistently, FOXO3 levels were higher in CTX‐injured TA muscles isolated from DBC1 knockdown mice (*Figure* *4* *B*). In contrast, knocking down DBC1 in proliferating C2C12 cells had no effect on FOXO3 protein levels (*Figure* *S8* *b*). These data indicated a negative correlation between DBC1 and FOXO3 protein levels during myogenesis. Meanwhile, knocking down FOXO3 in DBC1 knockdown C2C12 cells significantly increased the percentages of MyoG^+^ nuclei and MyoG protein level (*Figures* *4* *C* and *S9* *a*,*b*), as well as cell fusion rates and MHC protein levels (*Figures* *4* *D* and *S9* *c*). These results suggest that DBC1 regulates myogenesis by suppressing FOXO3. In addition to inhibiting myogenesis, FOXO3 was also reported to control myofibre wasting.^21^ In the muscles of aged mice, DBC1 protein decreased compared to those of the young (*Figure* *1* *A*), while FOXO3 protein levels increased (*Figure* *S10*). These data suggest that the negative correlation in DBC1 and FOXO3 proteins also exists in mature myofibres and that DBC1 might suppress myofibre wasting by inhibiting FOXO3. Consistent with this hypothesis, overexpressing DBC1 in fully differentiated myotubes decreased FOXO3 protein levels (*Figure* *4* *E*) and improved myotube quality as indicated by the stronger myotubes (*Figure* *4* *F*). These results indicate that DBC1 facilitates myogenesis and prevents myotube wasting by negatively regulating FOXO3.

### DBC1 deletion impairs mitochondrial functions due to increased FOXO3 during myogenesis

**Figure 5:**
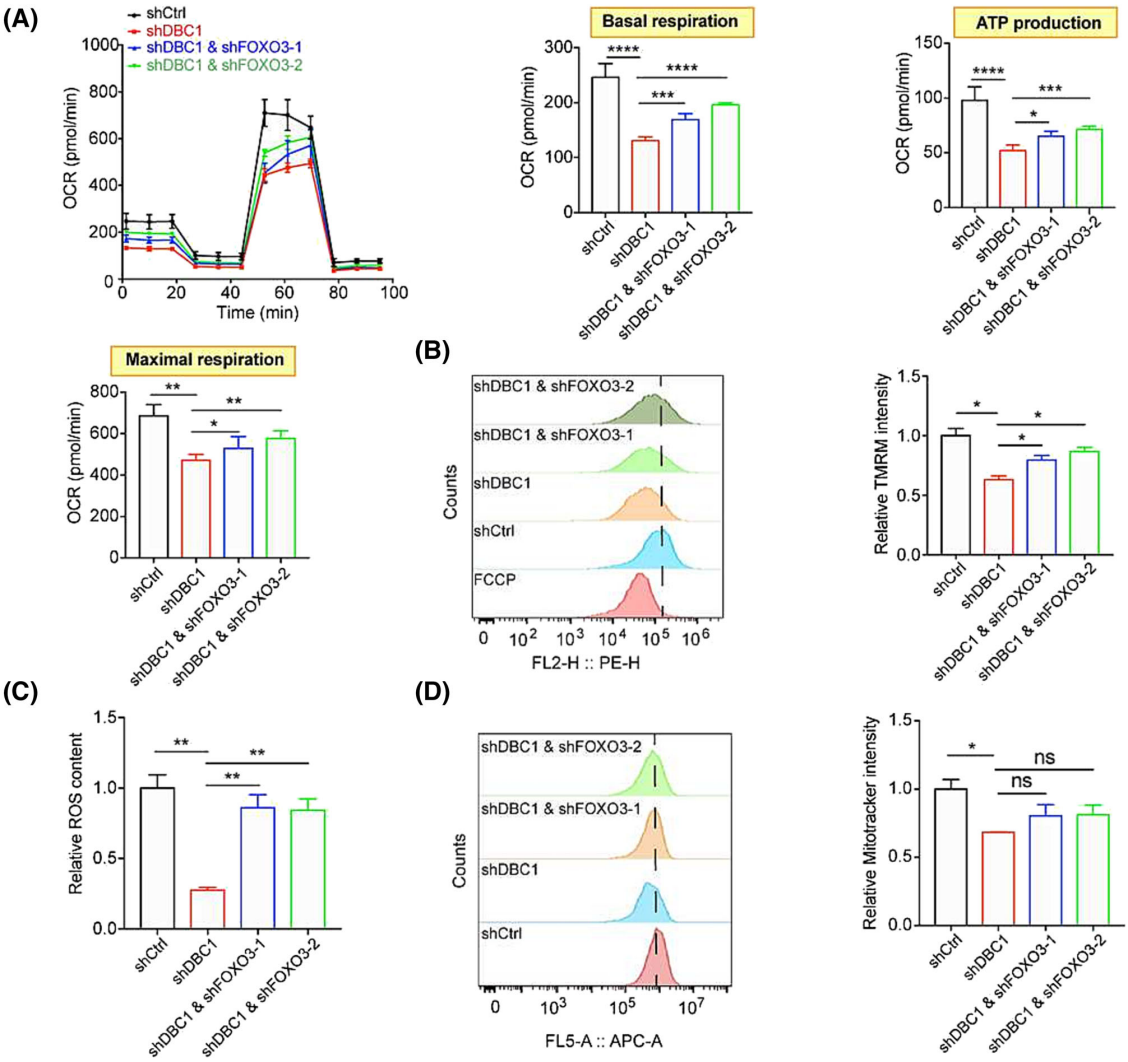
FOXO3 inhibits myogenesis by impairing mitochondrial functions. (A) Oxygen consumption rate (OCR) of DBC1 knockdown C2C12 cells, DBC1 and FOXO3 double knockdown C2C12 cells and the control cells that had been induced to differentiate for 2 days was performed to obtain bioenergetic parameters including basal respiration capacity, ATP‐linked OCR and maximal respiration, in the presence of oligomycin (Oligo) (10 μM), FCCP (40 μM), antimycin A (10 μM) and rotenone (10 μM), analysed by an extracellular flux analyser (Seahorse Bioscience). (B) (left) Mitochondrial membrane potential (MMP) of DBC1 knockdown C2C12 cells, DBC1 and FOXO3 double knockdown C2C12 cells and the control cells that had been induced to differentiate for 2 days, analysed by FACS. (right) Quantification of the MMP. (C) Measurements of ROS production of DBC1 knockdown C2C12 cells, DBC1 and FOXO3 double knockdown C2C12 cells and the control cells that had been induced to differentiate for 2 days, analysed by FACS. (D) (left) The number of mitochondria in DBC1 knockdown C2C12 cells, DBC1 and FOXO3 double knockdown C2C12 cells and the control cells that had been induced to differentiate for 2 days, analysed by FACS. (right) Quantification of the number of mitochondria. All *P* values were calculated using one‐way ANOVA for multiple comparison.

After demonstrating that DBC1 regulates myogenesis via suppressing FOXO3 (*Figure* *4* *C*,*D*), the question of how FOXO3 inhibits this process remains unanswered. FOXO3 has been reported to suppress mitochondrial functions.^29^ Mitochondria are linked to many factors that dictate the fate of satellite cells including metabolic reprograming upon activation,^30^ self‐renewal and differentiation.^S13,S14^ We hypothesized that DBC1 controlled myogenesis through FOXO3‐mediated regulation of mitochondrial functions. DBC1 knockdown reduced basal respiration, ATP production and maximal respiration of differentiating C2C12 cells measured by the Seahorse mitochondria stress test, while silencing FOXO3 in DBC1 knockdown cells reversed these declines (*Figure* *5* *A*). Consistent with the reduced mitochondrial respiratory function, mitochondrial membrane potential and reactive oxygen species (ROS) production were also decreased in differentiating DBC1 knockdown C2C12 cells, which could be rescued by silencing FOXO3 (*Figure* *5* *B*,*C*). The possibility that FOXO3 impairs mitochondrial function by decreasing the number of mitochondria was ruled out, as no difference was observed after knocking down FOXO3 in differentiating DBC1 knockdown C2C12 cells (*Figure* *5* *D*). However, no statistic difference was observed in mitochondrial membrane potential, mitochondria number, and ATP and ROS production in proliferating DBC1 knockdown C2C12 cells compared to the control cells (*Figure* *S11*). These results support the notion that elevated FOXO3 levels due to DBC1 deficiency hamper myogenesis through repressing mitochondrial functions.

### DBC1 knockdown accelerates myofibre wasting via FOXO3‐mediated ubiquitin–proteasome and autophagy–lysosome pathways

**Figure 6:**
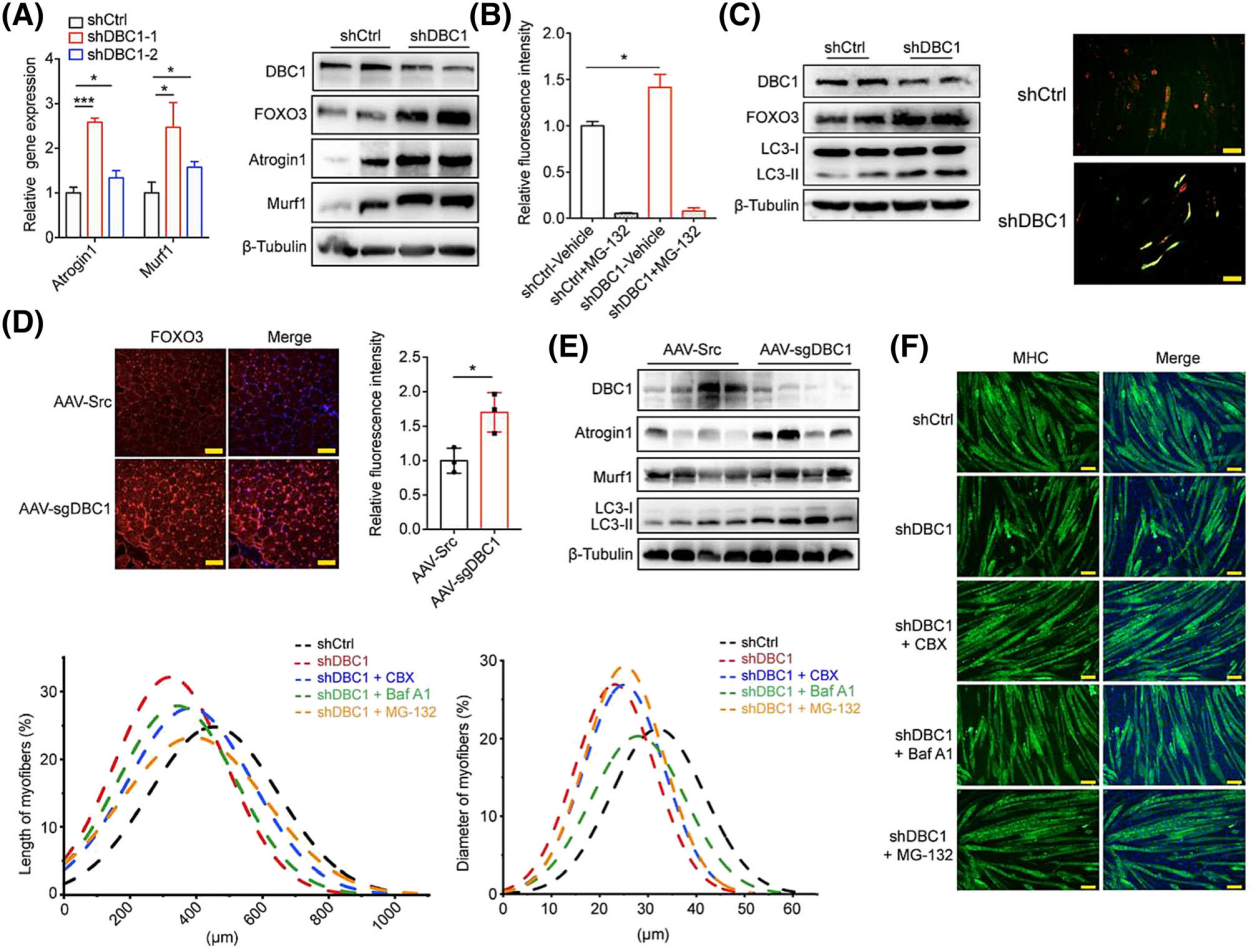
DBC1 knockdown accelerates myofibre wasting via FOXO3‐mediated activation of ubiquitin–proteasome pathway and autophagy–lysosome pathway. (A) (left) Relative gene expression of *Atrogin1* and *Murf1* in DBC1 knockdown and control myotubes, determined by RT‐qPCR. (right) Western blotting analysis for FOXO3, Atrogin1 and Murf1 protein levels in DBC1 knockdown and control myotubes. Before subjected to test, the myotubes had been induced to differentiate for 7 days followed by infection of lentivirus to knock down DBC1 for 2 days. (B) Proteasome activity in DBC1 knockdown or control myotubes. Proteasome activity was accessed in vitro using cell lysates of DBC1 knockdown and control myotubes that were treated as described in (A). MG‐132 (20 μM) was added in assay as a negative control. (C) (left) Western blotting analysis for FOXO3, LC3‐I and LC3‐II protein levels in DBC1 knockdown and control myotubes that were treated as described in (A). (right) Representative images of DBC1 knockdown and control myotubes transfected with autophagy reporter plasmid are shown on the right. Scale bar = 100 μm. (D) (left) Immunofluorescence staining of FOXO3 (red) in DBC1 knockdown TA muscles of mice as described in *Figure* *1* *C*. Nuclei were counterstained with DAPI (blue). Scale bars = 100 μm. (right) Quantification of fluorescence intensity (four mice for each group). (E) Western blotting analysis for Atrogin1, Murf1, LC3‐I and LC3‐II protein levels in TA muscles of DBC1 knockdown mice as described in *Figure* *1* *C*. (F) Immunofluorescence staining of MHC (green) in control myotubes, DBC1 knockdown myotubes and DBC1 knockdown myotubes treated by carbenoxolone (CBX, 100 μM), bafilomycin A1 (0.5 nM) and MG‐132 (10 nM), respectively. Nuclei were counterstained with DAPI (blue). Scale bar = 100 μm. And the myotube length and diameter were shown. *P* values were calculated using one‐way ANOVA for multiple comparison (A, B).

Next, we investigated how DBC1 regulated myofibre wasting. Given that FOXO3 regulates muscle atrophy by activating ubiquitin–proteasome pathway and autophagy–lysosome pathway,^11^ we first examined the expressions of Atrogin1 and Murf1, muscle‐specific ubiquitin ligases of ubiquitin–proteasome pathway, in DBC1 knockdown myotubes. We found that both Atrogin1 and Murf1 were upregulated at transcriptional and protein levels in DBC1 knockdown myotubes (*Figure* *6* *A*), along with significantly increased proteasome activity (*Figure* *6* *B*), suggesting activation of ubiquitin–proteasome pathway. Autophagy–lysosome pathway was also activated in DBC1 knockdown myotubes, as evidenced by increased conversion of LC3‐I to LC3‐II (*Figure* *6* *C*). This was confirmed by increased yellow puncta in DBC1 knockdown myotubes transfected with a fluorescent reporter plasmid to visualize autophagosomes (*Figure* *6* *C*). Consistently, we also found increased FOXO3 protein in mouse skeletal muscle with DBC1 ablation (*Figure* *6* *D*), as well as increased Atrogin1 and Murf1 and the conversion of LC3‐I to LC3‐II (*Figure* *6* *E*). Treating DBC1 knockdown myotubes with carbenoxolone, an inhibitor blocking FOXO3's transcriptional activity,^31^ decreased mRNA levels of *Atrogin1* and *Murf1* (*Figure* *S12* *a*) and improved the quality of myotubes (*Figure* *6* *F*). Autophagy inhibitor bafilomycin A1 and proteasome inhibitor MG‐132 achieved similar rescue effects on the quality of myotubes with DBC1 ablation (*Figure* *6* *F*). FOXO3 nuclear relocation is required for its transcriptional control of *Atrogin1* and *Murf1*, which is activated by FOXO3 dephosphorylation.^S15^ In line with the inhibitory effect of DBC1 on muscle atrophy, we found that DBC1 knockdown considerably increased dephosphorylation of FOXO3 during differentiated status and DBC1 overexpression did the opposite (*Figure* *S12* *b*,*c*). However, in the proliferating stage, phosphorylated FOXO3 increased, causing more FOXO3 relocated to cytoplasm (*Figure* *S13*). Together, these results support that DBC1 suppresses myotube wasting via FOXO3‐mediated activation of ubiquitin–proteasome and autophagy–lysosome pathways.

### DBC1 negatively regulates FOXO3 via ubiquitination–proteasome pathway

**Figure 7:**
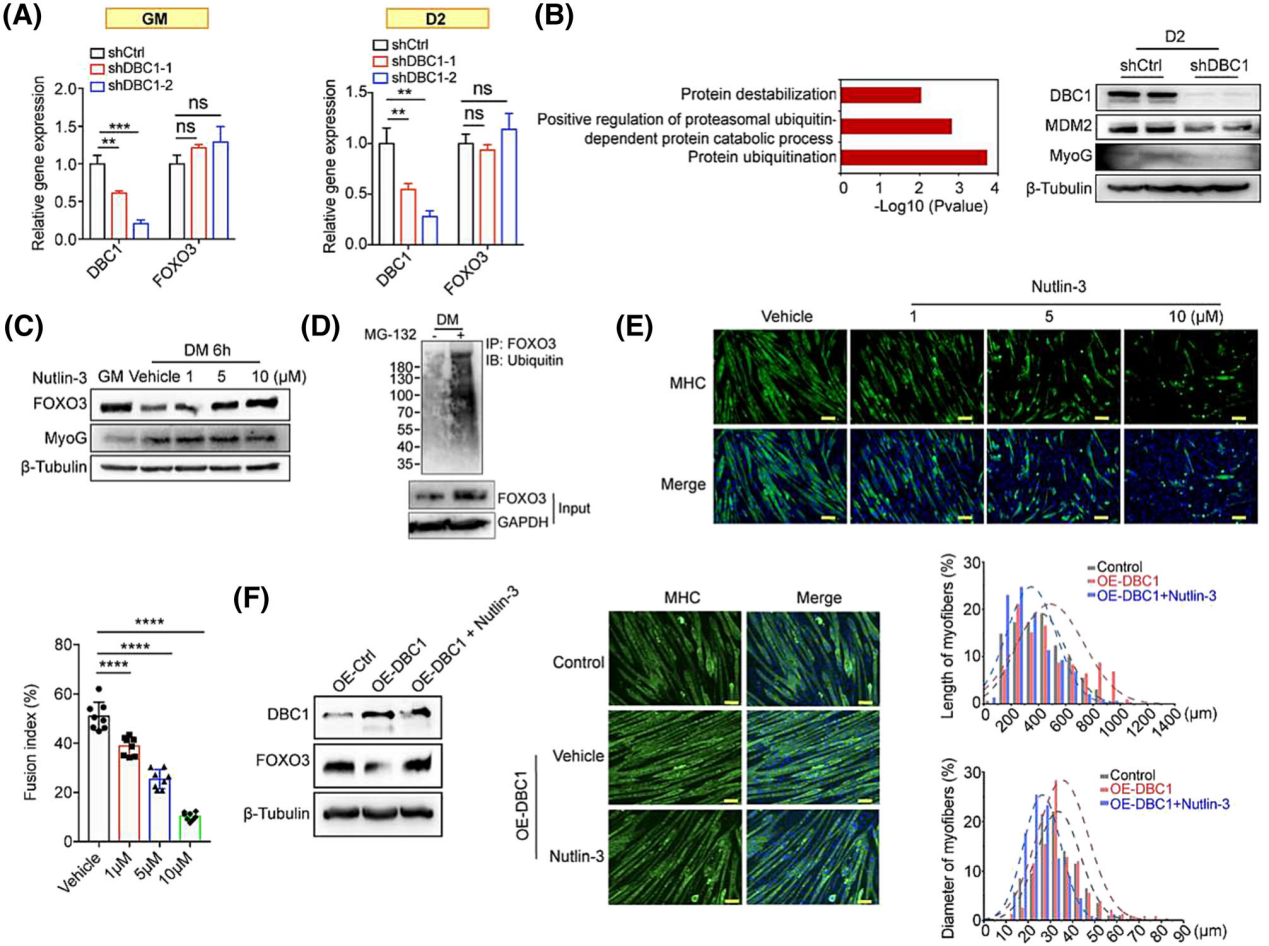
DBC1 promotes FOXO3 degradation via the ubiquitin–proteasome system. (A) Relative gene expression of *FOXO3* in proliferating (GM) (left) and differentiating (differentiate for 2 days [D2]) (right) DBC1 knockdown C2C12 cells and control cells, determined by RT‐qPCR. (B) (left) Gene Ontology (GO) analysis of the downregulated genes in DBC1 knockdown C2C12 cells, performed with −log_10_ (*P* value) plotted as a function of classification meeting a *P* value of <0.05. (right) Western blotting analysis for MDM2 and MyoG protein levels in DBC1 knockdown or control C2C12 cells that had been induced to differentiate for 2 days (D2). (C) Western blotting analysis for FOXO3 and MyoG protein levels in both proliferating C2C12 cells (GM) and C2C12 cells that had undergone differentiation for 6 h in the presence of Nutlin‐3 at 1‐, 5‐ and 10‐μM doses. (D) Western blotting analysis for FOXO3 ubiquitin levels in C2C12 cells that had been induced to differentiate for 3 h with the treatment of MG‐132 (1 μM) or not. (E) Immunofluorescence staining of MHC (green) in myotubes that were treated by Nutlin‐3 (at 1‐, 5‐ and 10‐μM doses) for the initial 24 h during differentiation process. The drug was then removed, and the differentiation continued until Day 7, at which point the samples were subjected to immunostaining. Nuclei were counterstained with DAPI (blue). Scale bar = 100 μm. The fusion index was quantified. (F) (left) Western blotting analysis for FOXO3 protein levels in DBC1 overexpression myotubes (differentiated for 7 days) with or without Nutlin‐3 (10 μM) treatment for 24 h. (right) Immunofluorescence staining of MHC (green) in DBC1 overexpression myotubes (differentiated for 7 days) with or without Nutlin‐3 (10 μM) treatment for 24 h. Nuclei were counterstained with DAPI (blue). Scale bar = 100 μm. Myotube length and diameter were quantified. *P* values were calculated using one‐way ANOVA for multiple comparison (A, E).

To further investigate how DBC1 modulates FOXO3, we first tested the mRNA expression of *FOXO3* but found that its transcription was not affected by DBC1 deletion in both proliferating and differentiating C2C12 cells (*Figure* *7* *A*). The GO analysis of the downregulated genes in DBC1 knockdown C2C12 cells revealed a significant enrichment of protein ubiquitination and protein destabilization (*Figure* *7* *B*), enlightening the possibility of FOXO3 degradation via ubiquitination upon differentiation. Previous studies reported that FOXO3 was subjected to MDM2‐mediated ubiquitination and proteasome degradation and that MDM2 knockdown raised FOXO3 protein levels.^32^ ^,^ ^S16^ We found that MDM2 protein was decreased after DBC1 knockdown in C2C12 cells (*Figures* *7* *B* and *S14*). Treatment of MDM2 inhibitor Nutlin‐3, or proteasome inhibitor MG‐132 and leupeptin blocked the reduction of FOXO3 during differentiation (*Figures* *7* *C* and *S15*), and the endogenously immunoprecipitated FOXO3 proteins from differentiating C2C12 cells were heavily ubiquitinated (*Figure* *7* *D*). Consistently, Nutlin‐3 significantly inhibited the differentiation of C2C12 cells, as indicated by decreased fusion rates (*Figure* *7* *E*). Similarly, high levels of DBC1 in myotubes could repress FOXO3 levels and improve myofibre quality, which can be reversed by Nutlin‐3 treatment (*Figure* *7* *F*). These data suggest that DBC1 controls ubiquitination‐mediated FOXO3 degradation by positively regulating MDM2 expression from early stages of myogenesis until fully differentiated stage.

## Discussion

In this study, we identified that DBC1 is essential for maintaining muscle mass by enhancing myogenesis and repressing myofibre wasting. Specifically, we found that DBC1 deletion in mouse skeletal muscle resulted in accelerated muscle loss, reduced muscle function and impaired muscle regeneration, all of which can be attributed to a common route of negatively regulating FOXO3. Further analysis showed that FOXO3 inhibited myogenesis by impairing mitochondrial functions and accelerated myofibre wasting through activating the ubiquitin–proteasome and autophagy–lysosome pathways. This study provides both molecular and functional evidences that DBC1 may represent a key regulator of skeletal muscle atrophy that is caused by physiological or pathological factors.

Although previous research has linked DBC1 deletion to a significant reduction of muscle function,^16^, ^17^ this study is the first to elucidate the mechanism by which DBC1 maintains the integrity of skeletal muscle via promoting myogenesis of satellite cells and repressing myofibre wasting. Our findings indicate that DBC1 is consistently found reduced in degenerating skeletal muscles due to a variety of causes including ageing, immobilization or pharmacological side effects, suggesting a common underlying mechanism. DBC1 negatively regulated MDM2‐mediated ubiquitination and degradation of FOXO3, which subsequently impaired mitochondrial functions during myogenesis and broke protein homeostasis in the mature myofibres. In adipose tissue, DBC1 has also been reported to have bifunctional roles in regulating adipocyte development and inflammation in fully differentiated 3T3‐L1 adipocytes.^33^ ^,^ ^S17^ These examples suggested that DBC1's function may vary depending on the development stages of cells. Most DBC1‐related research to date has been conducted in cells that are either mitosis‐competent or undifferentiated, so the role of DBC1 in terminally differentiated cells is poorly understood and requires further research in the future.

DBC1 is a known inhibitor of SIRT1, a key regulator of cellular functions such as metabolism, genomic stability and inflammation.^15^ Prior research has shown that SIRT1 activation has negative effects on myogenesis. For example, SIRT1 activity declined when quiescent satellite cells activate to achieve metabolic reprogramming from oxidation to glycolysis upon activation.^30^ SIRT1 activation mediated inhibition of myogenesis during glucose restriction^19^ and repressed myotube formation through suppressing MyoD transcription.^34^ Consistently, inhibition of SIRT1 activity by its inhibitor or hairpin RNA‐mediated knockdown accelerated myogenesis.^19^, ^30^ In our study, we found that DBC1 knockdown impaired myogenesis, which is consistent with impaired myogenesis upon SIRT1 activation. However, inhibiting SIRT1 activity by Ex‐527 or knocking down SIRT1 by hairpin RNA failed to rescue the impaired myogenesis (*Figure* *S7*), implying SIRT1 independence. Additionally, we found that DBC1 knockdown reduced mitochondrial functions, contrasting the previous finding that SIRT1 positively regulates mitochondrial biogenesis.^35^ These evidences ruled out the possibility that loss of DBC1‐impaired myogenesis was mediated by SIRT1 activation. Similarly, while SIRT1 has been shown to inhibit type I fibre atrophy during intermittent fasting^36^ and prevented myotube wasting in the presence of high glucose by maintaining expression of slower fibre type MHC,^37^ we found that DBC1 knockdown promoted myofibre wasting (*Figure* *3*). Together, these findings suggest that the effects of DBC1 on myogenesis and myofibre maintenance are independent of SIRT1.

FOXO3 has been identified as an important regulator in maintaining quiescence of satellite cells,^22^ although the underlying mechanisms are not yet fully understood. Mounting evidences suggest that mitochondria are important in controlling the fates of stem cells, including activation, self‐renewal and differentiation.^38^ ^,^ ^S18^ Mitochondrial dysfunctions have been shown to impair the myogenic capacity of satellite cells,^15^, ^16^ and inadequate mitochondrial activities resulting from ageing or genetic mutations can lead to failed myogenesis.^39^ ^,^ ^S19^ This study reported that mitochondrial functions were suppressed in DBC1 knockdown myoblasts, and this was ascribed to elevated FOXO3, which led to reduced oxidative phosphorylation capacity and impaired myogenesis. Reducing FOXO3 protein levels in DBC1 knockdown cells restored mitochondrial functions and myogenesis (*Figures* *4* *C*,*D* and *5*), demonstrating that FOXO3 regulates mitochondrial functions in DBC1‐regulated myogenesis. The precise mechanism by which FOXO3 regulates mitochondrial functions is an intriguing subject that will be investigated more in the future.

In fully differentiated myofibres, mitochondrial dysfunctions are frequently observed during myofibre wasting.^40^ ^,^ ^S20^ However, it is questionable whether there is a causality between mitochondrial dysfunctions and myofibre wasting, and if so, which one would be the cause. We also found that the metabolism was decreased in DBC1 knockdown myotubes (*Figure* *3* *D*–*F*), which is indicative of mitochondrial dysfunctions. Furthermore, our data revealed that the FOXO3 protein levels were elevated in DBC1 knockdown myotubes, and we observed upregulated Atrogin1 and Murf1 expression, as well as increased conversion of LC3‐I to LC3‐II (*Figure* *6* *A*–*C*). These findings are consistent with previous reports showing that FOXO3 upregulates atrophy‐related genes such as Atrogin1 and Mur1, as well as activates autophagy.^S21^ The degree of myofibre wasting was reduced in DBC1 knockdown cells by either blocking FOXO3's transcriptional activity or decreasing proteasome activity and autophagy flux, showing that FOXO3 mediates DBC1 ablation‐induced myofibre wasting via activating ubiquitin–proteasome pathway and autophagy–lysosome pathway.

Skeletal muscle atrophy is a significant health concern that poses a challenge for clinical treatment. This study assessed the role of DBC1 in skeletal muscle atrophy by regulating satellite cell myogenesis and myofibre wasting. These findings revealed a previously unknown function of DBC1 in counteracting skeletal muscle atrophy and provided new insight for potential therapeutic approaches to treat muscle atrophy.

## Conflict of interest statement

The authors report no conflicts of interest related to this work.

## Funding

This work was sponsored by CAMS Innovation Fund for Medical Sciences (2021‐I2M‐1‐050 and 2022‐I2M‐1‐012), National Key R&D Program of China (2022YFA1103803), the Non‐profit Central Research Institute Fund of Chinese Academy of Medical Sciences (2022‐JKCS‐14) and the State Key Laboratory Special Fund (2060204).

## Supporting information


**Figure S1.** Characterization of DBC1 protein levels in cachexia mice and DBC1 knockdown mice (a) Changes of body weight (tumor‐free) in cachexia mice. C57BL/6 (8 weeks old) male mice injected with 1.5 x 106 Lewis lung carcinoma (LLC) cells or vehicle (PBS). Skeletal muscles samples were collected 3 weeks after injection, *n* = 4 for each group. (b) Mass of skeletal muscles that normalized to body weight (n = 4 for each group). (c) Western blotting analysis for DBC1 protein levels in TA muscles isolated from mice described in (a). (d) Western blotting analysis for DBC1 protein levels in TA muscles isolated from DBC1 konckdown or the control mice. (e) Body weights of DBC1 konckdown and the control mice before sanction (*n* = 8 for each group). P values were calculated using two‐tailed Student's t‐test.

**Figure S2.** The regenerating TA muscle mass is decreased in DBC1 knockdown mice damaged by CTX (a) TA muscles weight of DBC1 knockdown or the control mice after 10 days of CTX damage (*n* = 4 for each group). (b) Representative images of TA muscles isolated from DBC1 knockdown and the control mice after 10 days of CTX damage. (c) Relative calf girth of DBC1 knockdown mice, normalized by values of the control mice after 10 days of CTX damage (*n* = 4 for each group). P values were calculated using two‐tailed Student's t‐test. (d‐e) Representative images of Immunofluorescence staining of MyoD (d) and MyoG (e) of TA muscles isolated from DBC1 knockdown or the control mice 10 days after CTX‐induced damage. Nuclei were counterstained with DAPI (blue). Arrowheads indicate representative cells express MyoD or MyoG. Scale bars = 100 μm.

**Figure S3.** DBC1 levels in the satellite cells are not affected by CTX injury (a) Schematic to illustrate experimental design of inducing TA muscles regeneration: TA muscles from 8‐week old C57BL / 6 J male mice were injected intramuscularly with 50 μL CTX (10 μM), followed by collecting muscle samples 5 days after the injection. (b) (Left) Immunofluorescence staining of Pax7 (green) and DBC1 (red) in TA muscles that were damaged by CTX for 5 days (injured) or not (uninjured). Nuclei were counterstained with DAPI (blue). Arrowheads indicate representative cells express DBC1 and Pax7 meanwhile. Scale bars = 100 μm. (Right) Percentages of DBC1‐ expressing Pax7 + progenitors measured by the ratio of Pax7+ / DBC1 + cells compared to Pax7 + progenitors. P values were calculated using two‐tailed Student's t‐test.

**Figure S4.** Differentially expressed genes in DBC1 knockdown C2C12 cells (a and b) Heat map (a) and volcano plot (b) of differentially expressed genes in DBC1 knockdown C2C12 cells.

**Figure S5.** DBC1 does not affect C2C12 cells proliferation and apoptosis (a) (Left) Immunofluorescence staining of Ki67 (red) in proliferating DBC1 knockdown and the control C2C12 cells. Nuclei were counterstained with DAPI (blue). Scale bars = 100 μm. (Right) Percentages of Ki67 positive (Ki67+) nuclei. (b) (Left) Representative scatter plots of apoptotic analysis of proliferating DBC1 knockdown and the control C2C12 cells using Annexin V‐FITC staining and flow cytometry. (Right) Quantification of the proportion of apoptotic cells. P values were calculated using oneway ANOVA for multiple comparison.

**Figure S6.** DBC1 overexpression attenuates muscle atrophy (a) Western blotting analysis for DBC1 protein levels in DBC1 knockdown and the control myotubes. C2C12 cells were induced to fully differentiate for 7 days, followed y adding lentivirus to knock down DBC1 for 48 h. (b) Western blotting analysis for DBC1 protein level in serum fast induced atrophy myotubes and the control myotubes. C2C12 cells were fully differentiated for 7 days and then subjected to serum fast for 24 h. (c) Western blotting analysis for DBC1 protein levels in DBC1 overexpression and the control myotubes. C2C12 cells were fully differentiated for 7 days, followed by adding retrovirus to overexpress DBC1 for 48 h. (d) (Left) Immunofluorescence staining of MHC (green) in DBC1 overexpression and the control myotubes described in (c) that were either serum fasted for 24 h or not. Nuclei were counterstained with DAPI (blue), scale bar = 100 μm. (Right) Myotubes length and diameter were quantified. (e‐f) Western blotting analysis for DBC1, Atrogin1 and Murf1 protein levels in the muscles of old mice (e) and mice with muscle atrophy induced by limb immobilization (f) that were injected with retrovirus overexpressing DBC1. Old mice (12 months) were injected once with retrovirus and then sanctioned 10 days later. Young mice (3 months) were subjected to right limb immobilization for 2 weeks, with retrovirus injections on days 1 and 8.

**Figure S7.** DBC1 regulates myogenesis independent of SIRT1 (a) (Left) Immunofluorescence staining of MyoG (green) in DBC1 knockdown and the control C2C12 cells that had been induced to differentiate for 2 days with the treatment of Ex‐527 (10 μM) or not. Nuclei were counterstained with DAPI (blue). Scale bars = 100 μm. (Right) Quantification of the proportion of MyoG+ nuclei. (b) (Left) Immunofluorescence staining of MHC (green) in DBC1 knockdown and the control C2C12 cells that had been induced to differentiate for 7 days with the treatment of Ex‐ 527 (10 μM) or not. Nuclei were counterstained with DAPI (blue). Scale bars = 100 μm. (Right) Quantification of the fusion index. (c) Relative gene expression of *SIRT1* in DBC1 knockdown C2C12 cells that were added lentivirus to knock down SIRT1 for 48 h, determined by RT‐qPCR. (d) (Left) Immunofluorescence staining of MyoG (green) in DBC1 knockdown, DBC1 and SIRT1 double knockdown and the control C2C12 cells that had been induced to differentiate for 2 days. Nuclei were counterstained with DAPI (blue). Scale bars = 100 μm. (Right) Quantification of the proportion of MyoG+ nuclei. (e) (Left) Immunofluorescence staining of MHC (green) in DBC1 knockdown, DBC1 and SIRT1 double knockdown and the control C2C12 cells that had been induced to differentiate for 7 days. Nuclei were counterstained with DAPI (blue). Scale bars = 100 μm. (Right) Quantification of the fusion index. P values were calculated using one‐way ANOVA for multiple comparison.

**Figure S8.** DBC1 negatively regulates FOXO3 during myogenesis (a) Western blotting analysis for FOXO3 and MyoG protein levels in DBC1 knockdown C2C12 cells, DBC1 knockdown C2C12 cells that re‐expressed DBC1 or the control cells that had been induced to differentiate for 2 days (D2). (b) Western blotting analysis for FOXO3 protein levels in proliferating DBC1 knockdown and the control C2C12 cells.

**Figure S9.** FOXO3 deletion in DBC1 knockdown C2C12 cells rescues myogenesis (a) Western blotting analysis for FOXO3 protein levels in DBC1 knockdown C2C12 cells that were added lentivirus to knock down FOXO3 for 48 h. (b and c) Western lotting analysis for MyoG protein levels (b) and MHC protein levels (c) in DBC1 knockdown C2C12 cells, DBC1 and FOXO3 double knockdown C2C12 cells and the control cells that had been induced to differentiate for 2 days and 7 days, respectively.

**Figure S10.** FOXO3 expression is increased in old skeletal muscle (a) Immunofluorescence staining of FOXO3 (red) in TA muscles isolated from young (3 months) and old (24 months) C57BL / 6 J mice. Nuclei were counterstained with DAPI (blue). Scale bars = 100 μm. (b) Western blotting analysis for FOXO3 protein levels in TA muscles from young (3 months) and old (24 months) C57BL / 6 J mice. P values were calculated using two‐tailed Student's t‐test.

**Figure S11.** DBC1 knockdown does not affect mitochondrial functions in proliferating C2C12 cells (a) Mitochondrial membrane potential (MMP) of proliferating DBC1 knockdown and the control C2C12 cells that were analyzed by FACS. (b) The number of mitochondria of proliferating DBC1 knockdown and the control C2C12 cells were analyzed by FACS. (c) ATP production was accessed in vitro using cell lysates of proliferating DBC1 knockdown and the control C2C12 cells. (d) Measurements of ROS production of proliferating DBC1 knockdown and the control C2C12 cells, analyzed by FACS. P values were calculated using one‐way ANOVA for multiple comparison.

**Figure S12.** DBC1 negatively regulates the transcriptional activity and dephosphorylation of FOXO3 in differentiated cells (a) Relative gene expression of *Atrogin1* and *Murf1* in DBC1 knockdown myotubes, DBC1 knockdown myotubes treated by Carbenoxolone (CBX, 100 μM) and the control myotubes, determined by RT‐qPCR. (b‐c) Western blotting analysis for phosphorylated FOXO3 (p‐FOXO3) and total FOXO3 protein levels in DBC1 knockdown (b) and DBC1 overexpression (c) myotubes. The myotubes were fully differentiated for 7 days before infected for 2 days with lentiviruses that knocked down DBC1 or retroviruses that overexpressed DBC1. P values were calculated using one‐way ANOVA for multiple comparison.

**Figure S13.** DBC1 negatively regulates the phosphorylation of FOXO3 in proliferating C2C12 cells (a) Western blotting analysis for phosphorylated FOXO3 (p‐FOXO3) and total FOXO3 protein levels in DBC1 knockdown and the control proliferating C2C12 cells. (b) (Left) Representative images of Immunofluorescence staining of FOXO3 (red) in DBC1 knockdown and the control proliferating C2C12 cells. Nuclei were counterstained with DAPI (blue). Scale bars = 400 μm. (Right) Quantification of the nuclear / cytoplasmic fluorescence ratio. P values were calculated using oneway ANOVA for multiple comparison.

**Figure S14.** DBC1 positively regulates MDM2 expression (a) Relative gene expression of *MDM2* in proliferating DBC1 knockdown and the control C2C12 cells, determined by RT‐qPCR. (b) Western blotting analysis for MDM2 protein levels in proliferating DBC1 knockdown C2C12 cells and the control cells. P values were calculated using two‐tailed Student's t‐test.

**Figure S15.** Ubiquitinated FOXO3 is degraded via proteasome pathway (a and b) Western blotting analysis for FOXO3 and MyoG protein levels in C2C12 cells that had been induced to differentiate for 0 h, 3 h or 6 h with the treatment of MG‐ 132 (1 μM) (a) or Leupeptin (10 μM) (b).

**Data S1.** Supporting Information

## Data Availability

All data are available in the main text or the supporting information.
