# Supplementary material for: DBC1 maintains skeletal muscle integrity by enhancing myogenesis and preventing myofibre wasting
Source: J Cachexia Sarcopenia Muscle. 2023 Dec 7;15(1):255–69. doi: 10.1002/jcsm.13398 (PMC10834312; doi:10.1002/jcsm.13398)
Supplement: Supplementary file 1 — Figure S1. Characterization of DBC1 protein levels in cachexia mice and DBC1 knockdown mice (a) Changes of body weight (tumor‐free) in cachexia mice. C57BL/6 (8 weeks old) male mice injected with 1.5 x 10^6^ Lewis lung carcinoma (LLC) cells or vehicle (PBS). Skeletal muscles samples were collected 3 weeks after injection, n = 4 for each group. (b) Mass of skeletal muscles that normalized to body weight (n = 4 for each group). (c) Western blotting analysis for DBC1 protein levels in TA muscles isolated from mice described in (a). (d) Western blotting analysis for DBC1 protein levels in TA muscles isolated from DBC1 konckdown or the control mice. (e) Body weights of DBC1 konckdown and the control mice before sanction (n = 8 for each group). P values were calculated using two‐tailed Student's t‐test. [file JCSM-15-255-s012.pdf]

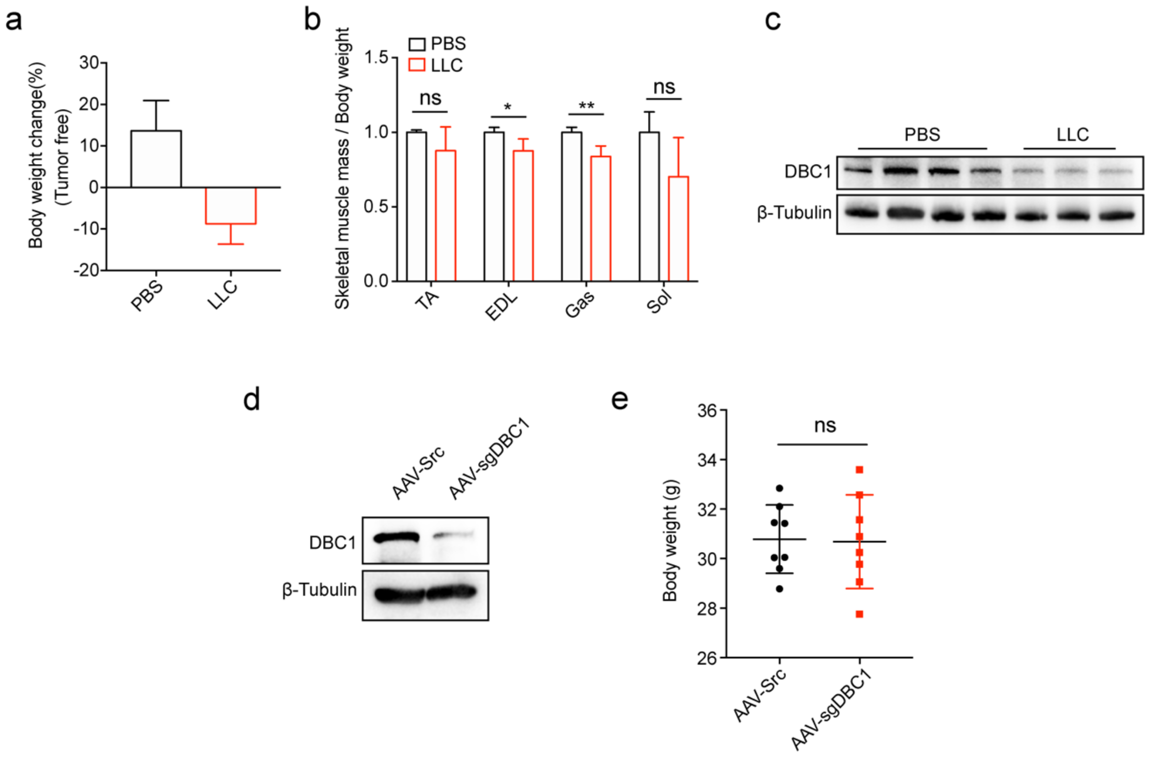

Supplementary Fig. 1 Characterization of DBC1 protein levels in cachexia mice and DBC1 knockdown mice

(a) Changes of body weight (tumor-free) in cachexia mice. C57BL/6 (8 weeks old) male mice injected with 1.5 x 10^6 Lewis lung carcinoma (LLC) cells or vehicle (PBS). Skeletal muscles samples were collected 3 weeks after injection, n = 4 for each group. **(b)** Mass of skeletal muscles that normalized to body weight (n = 4 for each group). **(c)** Western blotting analysis for DBC1 protein levels in TA muscles isolated from mice described in (a). **(d)** Western blotting analysis for DBC1 protein levels in TA muscles isolated from DBC1 konckdown or the control mice. **(e)** Body weights of DBC1 konckdown and the control mice before sanction (n = 8 for each group). P values were calculated using two-tailed Student's t-test.
