# Supplementary material for: DBC1 maintains skeletal muscle integrity by enhancing myogenesis and preventing myofibre wasting
Source: J Cachexia Sarcopenia Muscle. 2023 Dec 7;15(1):255–69. doi: 10.1002/jcsm.13398 (PMC10834312; doi:10.1002/jcsm.13398)
Supplement: Supplementary file 2 — Figure S2. The regenerating TA muscle mass is decreased in DBC1 knockdown mice damaged by CTX (a) TA muscles weight of DBC1 knockdown or the control mice after 10 days of CTX damage (n = 4 for each group). (b) Representative images of TA muscles isolated from DBC1 knockdown and the control mice after 10 days of CTX damage. (c) Relative calf girth of DBC1 knockdown mice, normalized by values of the control mice after 10 days of CTX damage (n = 4 for each group). P values were calculated using two‐tailed Student's t‐test. (d‐e) Representative images of Immunofluorescence staining of MyoD (d) and MyoG (e) of TA muscles isolated from DBC1 knockdown or the control mice 10 days after CTX‐induced damage. Nuclei were counterstained with DAPI (blue). Arrowheads indicate representative cells express MyoD or MyoG. Scale bars = 100 μm. [file JCSM-15-255-s008.pdf]

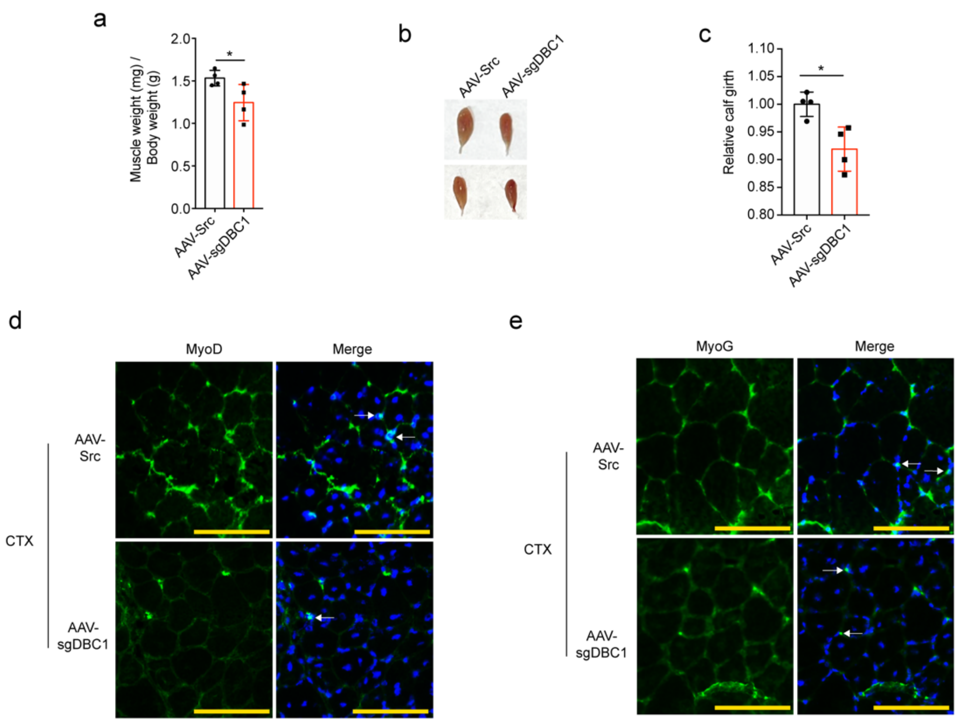

Supplementary Fig. 2 The regenerating TA muscle mass is decreased in DBC1 knockdown mice damaged by CTX

(a) TA muscles weight of DBC1 knockdown or the control mice after 10 days of CTX damage (n = 4 for each group). **(b)** Representative images of TA muscles isolated from DBC1 knockdown and the control mice after 10 days of CTX damage. **(c)** Relative calf girth of DBC1 knockdown mice, normalized by values of the control mice after 10 days of CTX damage (n = 4 for each group). P values were calculated using two-tailed Student's t-test. **(d-e) Representative images of Immunofluorescence staining of MyoD (d) and MyoG (e) of TA muscles isolated from DBC1 knockdown or the control mice 10 days after CTX-induced damage. Nuclei were counterstained with DAPI (blue). Arrowheads indicate representative cells express MyoD or MyoG. Scale bars = 100 μm.**
