# Supplementary material for: DBC1 maintains skeletal muscle integrity by enhancing myogenesis and preventing myofibre wasting
Source: J Cachexia Sarcopenia Muscle. 2023 Dec 7;15(1):255–69. doi: 10.1002/jcsm.13398 (PMC10834312; doi:10.1002/jcsm.13398)
Supplement: Supplementary file 3 — Figure S3. DBC1 levels in the satellite cells are not affected by CTX injury (a) Schematic to illustrate experimental design of inducing TA muscles regeneration: TA muscles from 8‐week old C57BL / 6 J male mice were injected intramuscularly with 50 μL CTX (10 μM), followed by collecting muscle samples 5 days after the injection. (b) (Left) Immunofluorescence staining of Pax7 (green) and DBC1 (red) in TA muscles that were damaged by CTX for 5 days (injured) or not (uninjured). Nuclei were counterstained with DAPI (blue). Arrowheads indicate representative cells express DBC1 and Pax7 meanwhile. Scale bars = 100 μm. (Right) Percentages of DBC1‐ expressing Pax7 + progenitors measured by the ratio of Pax7+ / DBC1 + cells compared to Pax7 + progenitors. P values were calculated using two‐tailed Student's t‐test. [file JCSM-15-255-s015.pdf]

a

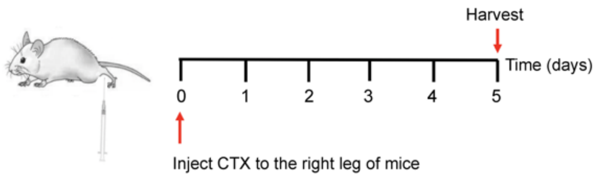

b

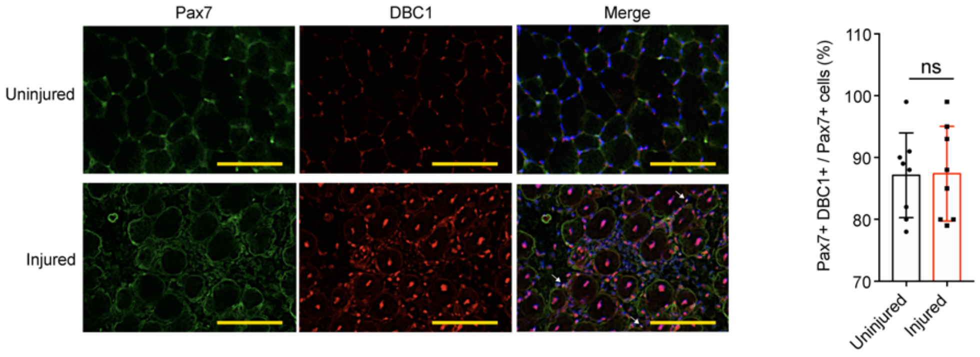

Supplementary Fig. 3 DBC1 levels in the satellite cells are not affected by CTX injury

(a) Schematic to illustrate experimental design of inducing TA muscles regeneration: TA muscles from 8-week old C57BL / 6J male mice were injected intramuscularly with 50 μL CTX (10 μM), followed by collecting muscle samples 5 days after the injection. **(b)** (Left) Immunofluorescence staining of Pax7 (green) and DBC1 (red) in TA muscles that were damaged by CTX for 5 days (injured) or not (uninjured). Nuclei were counterstained with DAPI (blue). Arrowheads indicate representative cells express DBC1 and Pax7 meanwhile. Scale bars = 100 μm. (Right) Percentages of DBC1-expressing $Pax7^+$ progenitors measured by the ratio of $Pax7^+$ / $DBC1^+$ cells compared to $Pax7^+$ progenitors. P values were calculated using two-tailed Student's t-test.
