# Supplementary material for: DBC1 maintains skeletal muscle integrity by enhancing myogenesis and preventing myofibre wasting
Source: J Cachexia Sarcopenia Muscle. 2023 Dec 7;15(1):255–69. doi: 10.1002/jcsm.13398 (PMC10834312; doi:10.1002/jcsm.13398)
Supplement: Supplementary file 4 — Figure S4. Differentially expressed genes in DBC1 knockdown C2C12 cells (a and b) Heat map (a) and volcano plot (b) of differentially expressed genes in DBC1 knockdown C2C12 cells. [file JCSM-15-255-s013.pdf]

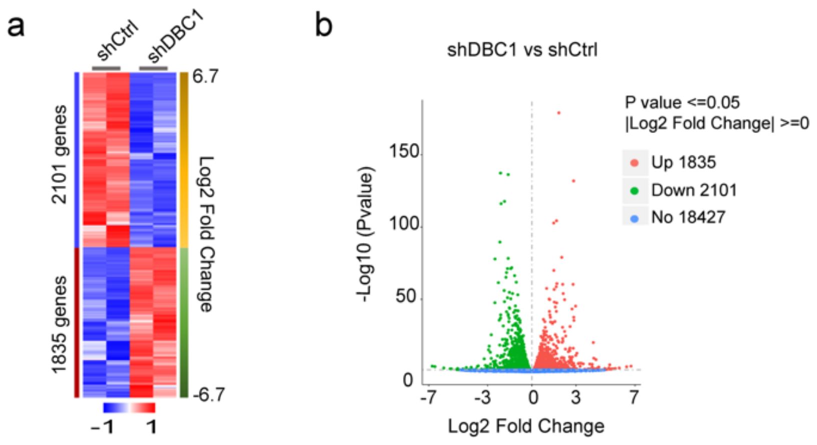

Supplementary Fig. 4 Differentially expressed genes in DBC1 knockdown C2C12 cells

(a and b) Heat map (a) and volcano plot (b) of differentially expressed genes in DBC1 knockdown C2C12 cells.
