# Supplementary material for: DBC1 maintains skeletal muscle integrity by enhancing myogenesis and preventing myofibre wasting
Source: J Cachexia Sarcopenia Muscle. 2023 Dec 7;15(1):255–69. doi: 10.1002/jcsm.13398 (PMC10834312; doi:10.1002/jcsm.13398)
Supplement: Supplementary file 5 — Figure S5. DBC1 does not affect C2C12 cells proliferation and apoptosis (a) (Left) Immunofluorescence staining of Ki67 (red) in proliferating DBC1 knockdown and the control C2C12 cells. Nuclei were counterstained with DAPI (blue). Scale bars = 100 μm. (Right) Percentages of Ki67 positive (Ki67+) nuclei. (b) (Left) Representative scatter plots of apoptotic analysis of proliferating DBC1 knockdown and the control C2C12 cells using Annexin V‐FITC staining and flow cytometry. (Right) Quantification of the proportion of apoptotic cells. P values were calculated using oneway ANOVA for multiple comparison. [file JCSM-15-255-s003.pdf]

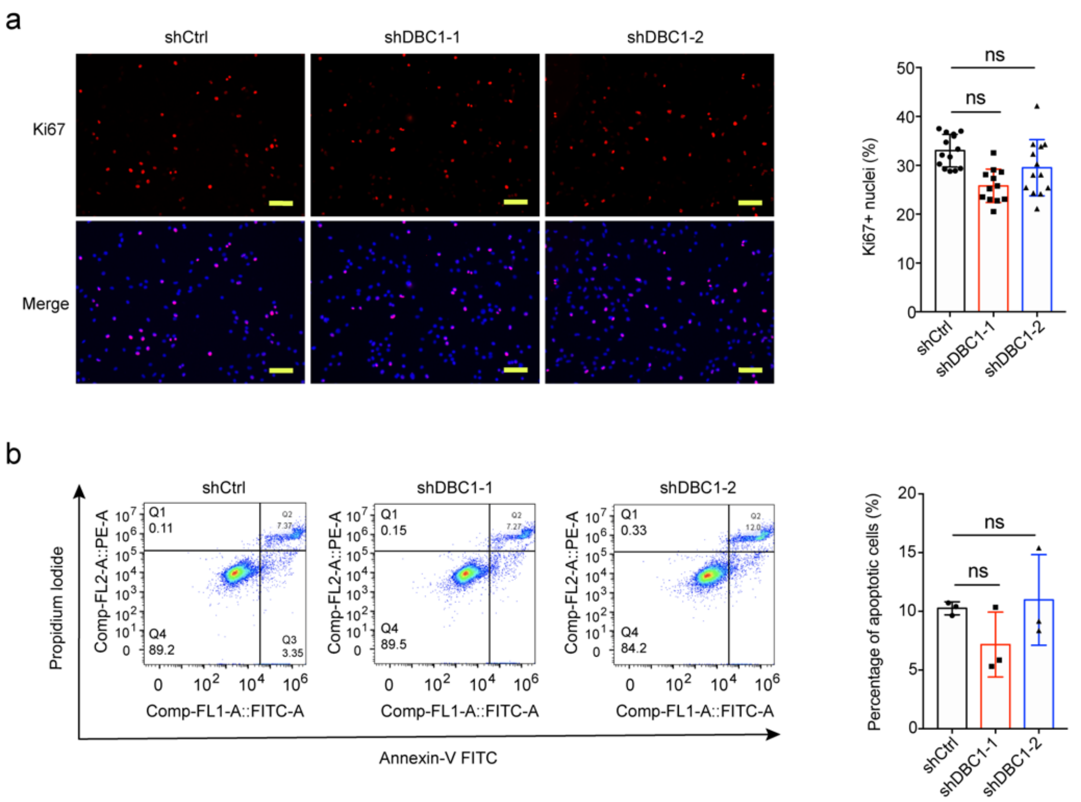

Supplementary Fig. 5 DBC1 does not affect C2C12 cells proliferation and apoptosis

(a) (Left) Immunofluorescence staining of Ki67 (red) in proliferating DBC1 knockdown and the control C2C12 cells. Nuclei were counterstained with DAPI (blue). Scale bars = 100 µm. (Right) Percentages of Ki67 positive ($Ki67^{+}$) nuclei. **(b)** (Left) Representative scatter plots of apoptotic analysis of proliferating DBC1 knockdown and the control C2C12 cells using Annexin V-FITC staining and flow cytometry. (Right) Quantification of the proportion of apoptotic cells. P values were calculated using one-way ANOVA for multiple comparison.
