# Supplementary material for: DBC1 maintains skeletal muscle integrity by enhancing myogenesis and preventing myofibre wasting
Source: J Cachexia Sarcopenia Muscle. 2023 Dec 7;15(1):255–69. doi: 10.1002/jcsm.13398 (PMC10834312; doi:10.1002/jcsm.13398)
Supplement: Supplementary file 6 — Figure S6. DBC1 overexpression attenuates muscle atrophy (a) Western blotting analysis for DBC1 protein levels in DBC1 knockdown and the control myotubes. C2C12 cells were induced to fully differentiate for 7 days, followed y adding lentivirus to knock down DBC1 for 48 h. (b) Western blotting analysis for DBC1 protein level in serum fast induced atrophy myotubes and the control myotubes. C2C12 cells were fully differentiated for 7 days and then subjected to serum fast for 24 h. (c) Western blotting analysis for DBC1 protein levels in DBC1 overexpression and the control myotubes. C2C12 cells were fully differentiated for 7 days, followed by adding retrovirus to overexpress DBC1 for 48 h. (d) (Left) Immunofluorescence staining of MHC (green) in DBC1 overexpression and the control myotubes described in (c) that were either serum fasted for 24 h or not. Nuclei were counterstained with DAPI (blue), scale bar = 100 μm. (Right) Myotubes length and diameter were quantified. (e‐f) Western blotting analysis for DBC1, Atrogin1 and Murf1 protein levels in the muscles of old mice (e) and mice with muscle atrophy induced by limb immobilization (f) that were injected with retrovirus overexpressing DBC1. Old mice (12 months) were injected once with retrovirus and then sanctioned 10 days later. Young mice (3 months) were subjected to right limb immobilization for 2 weeks, with retrovirus injections on days 1 and 8. [file JCSM-15-255-s010.pdf]

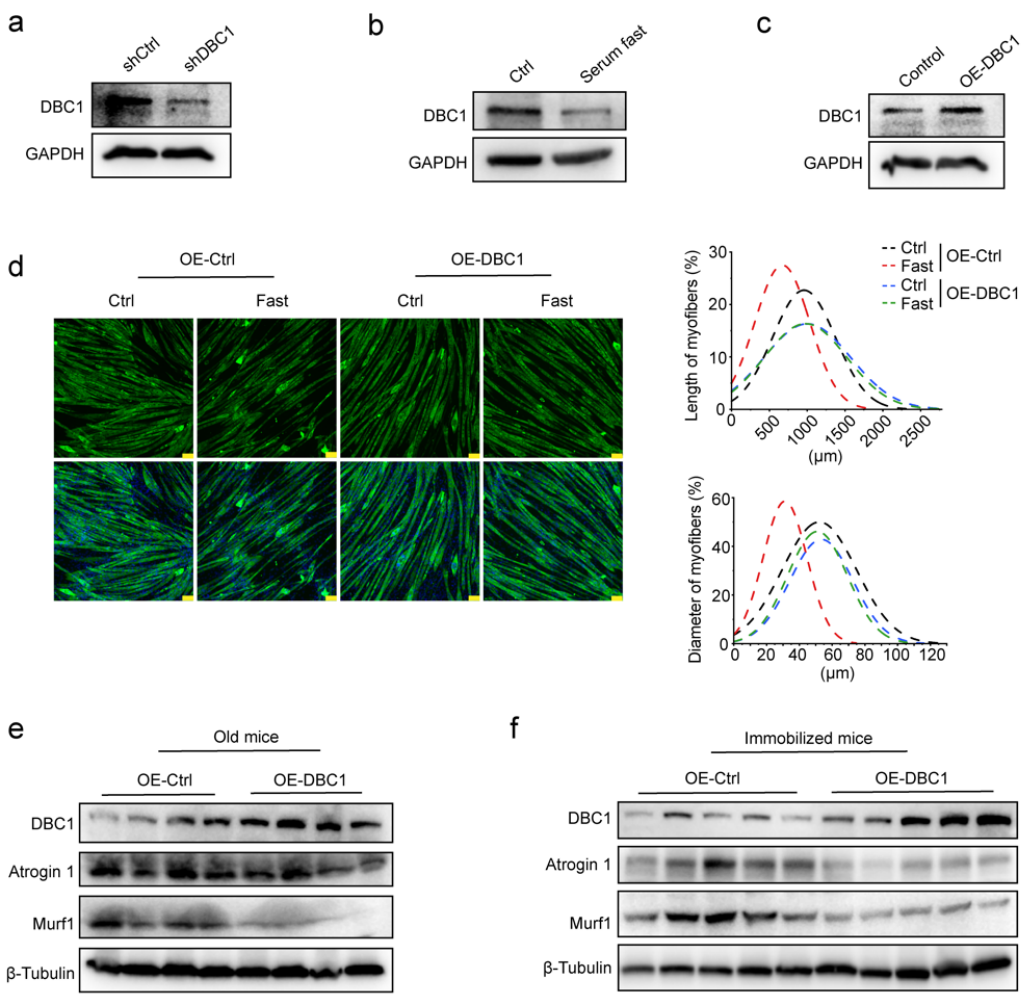

Supplementary Fig. 6 DBC1 overexpression attenuates muscle atrophy

(a) Western blotting analysis for DBC1 protein levels in DBC1 knockdown and the control myotubes. C2C12 cells were induced to fully differentiate for 7 days, followed by adding lentivirus to knock down DBC1 for 48 h. **(b)** Western blotting analysis for DBC1 protein level in serum fast induced atrophy myotubes and the control myotubes. C2C12 cells were fully differentiated for 7 days and then subjected to serum fast for 24h. **(c)** Western blotting analysis for DBC1 protein levels in DBC1 overexpression and the control myotubes. C2C12 cells were fully differentiated for 7 days, followed by adding retrovirus to overexpress DBC1 for 48 h. **(d)** (Left) Immunofluorescence

staining of MHC (green) in DBC1 overexpression and the control myotubes described in (c) that were either serum fasted for 24 h or not. Nuclei were counterstained with DAPI (blue), scale bar = 100 μm. (Right) Myotubes length and diameter were quantified. **(e-f)** Western blotting analysis for DBC1, Atrogin1 and Murf1 protein levels in the muscles of old mice (e) and mice with muscle atrophy induced by limb immobilization (f) that were injected with retrovirus overexpressing DBC1. Old mice (12 months) were injected once with retrovirus and then sanctioned 10 days later. Young mice (3 months) were subjected to right limb immobilization for 2 weeks, with retrovirus injections on days 1 and 8.
