# Supplementary material for: DBC1 maintains skeletal muscle integrity by enhancing myogenesis and preventing myofibre wasting
Source: J Cachexia Sarcopenia Muscle. 2023 Dec 7;15(1):255–69. doi: 10.1002/jcsm.13398 (PMC10834312; doi:10.1002/jcsm.13398)
Supplement: Supplementary file 7 — Figure S7. DBC1 regulates myogenesis independent of SIRT1 (a) (Left) Immunofluorescence staining of MyoG (green) in DBC1 knockdown and the control C2C12 cells that had been induced to differentiate for 2 days with the treatment of Ex‐527 (10 μM) or not. Nuclei were counterstained with DAPI (blue). Scale bars = 100 μm. (Right) Quantification of the proportion of MyoG+ nuclei. (b) (Left) Immunofluorescence staining of MHC (green) in DBC1 knockdown and the control C2C12 cells that had been induced to differentiate for 7 days with the treatment of Ex‐ 527 (10 μM) or not. Nuclei were counterstained with DAPI (blue). Scale bars = 100 μm. (Right) Quantification of the fusion index. (c) Relative gene expression of SIRT1 in DBC1 knockdown C2C12 cells that were added lentivirus to knock down SIRT1 for 48 h, determined by RT‐qPCR. (d) (Left) Immunofluorescence staining of MyoG (green) in DBC1 knockdown, DBC1 and SIRT1 double knockdown and the control C2C12 cells that had been induced to differentiate for 2 days. Nuclei were counterstained with DAPI (blue). Scale bars = 100 μm. (Right) Quantification of the proportion of MyoG+ nuclei. (e) (Left) Immunofluorescence staining of MHC (green) in DBC1 knockdown, DBC1 and SIRT1 double knockdown and the control C2C12 cells that had been induced to differentiate for 7 days. Nuclei were counterstained with DAPI (blue). Scale bars = 100 μm. (Right) Quantification of the fusion index. P values were calculated using one‐way ANOVA for multiple comparison. [file JCSM-15-255-s007.pdf]

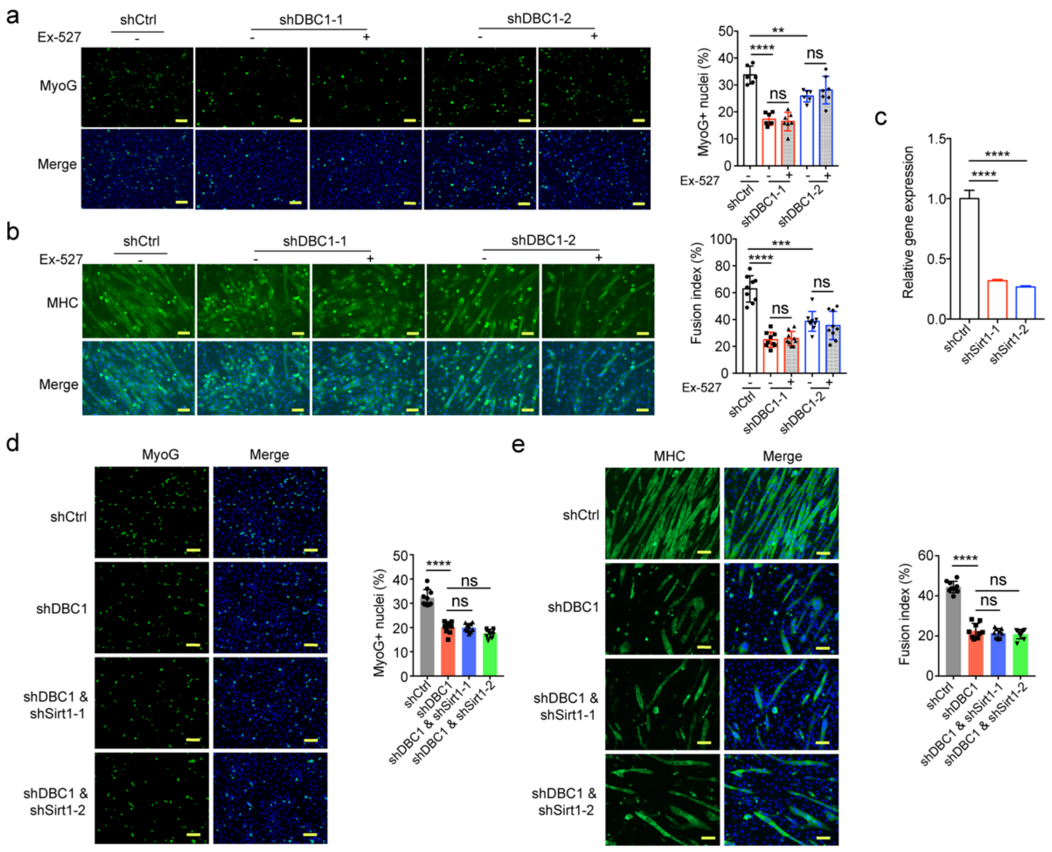

Supplementary Fig. 7 DBC1 regulates myogenesis independent of SIRT1

(a) (Left) Immunofluorescence staining of MyoG (green) in DBC1 knockdown and the control C2C12 cells that had been induced to differentiate for 2 days with the treatment of Ex-527 (10 µM) or not. Nuclei were counterstained with DAPI (blue). Scale bars = 100 µm. (Right) Quantification of the proportion of MyoG^{+} nuclei. **(b)** (Left) Immunofluorescence staining of MHC (green) in DBC1 knockdown and the control C2C12 cells that had been induced to differentiate for 7 days with the treatment of Ex-527 (10 µM) or not. Nuclei were counterstained with DAPI (blue). Scale bars = 100 µm. (Right) Quantification of the fusion index. **(c)** Relative gene expression of *SIRT1* in DBC1 knockdown C2C12 cells that were added lentivirus to knock down SIRT1 for 48 h, determined by RT-qPCR. **(d)** (Left) Immunofluorescence staining of MyoG (green)

in DBC1 knockdown, DBC1 and SIRT1 double knockdown and the control C2C12 cells that had been induced to differentiate for 2 days. Nuclei were counterstained with DAPI (blue). Scale bars = 100 μm. (Right) Quantification of the proportion of $MyoG^+$ nuclei. **(e)** (Left) Immunofluorescence staining of MHC (green) in DBC1 knockdown, DBC1 and SIRT1 double knockdown and the control C2C12 cells that had been induced to differentiate for 7 days. Nuclei were counterstained with DAPI (blue). Scale bars = 100 μm. (Right) Quantification of the fusion index. P values were calculated using one-way ANOVA for multiple comparison.
