# Supplementary material for: DBC1 maintains skeletal muscle integrity by enhancing myogenesis and preventing myofibre wasting
Source: J Cachexia Sarcopenia Muscle. 2023 Dec 7;15(1):255–69. doi: 10.1002/jcsm.13398 (PMC10834312; doi:10.1002/jcsm.13398)
Supplement: Supplementary file 8 — Figure S8. DBC1 negatively regulates FOXO3 during myogenesis (a) Western blotting analysis for FOXO3 and MyoG protein levels in DBC1 knockdown C2C12 cells, DBC1 knockdown C2C12 cells that re‐expressed DBC1 or the control cells that had been induced to differentiate for 2 days (D2). (b) Western blotting analysis for FOXO3 protein levels in proliferating DBC1 knockdown and the control C2C12 cells. [file JCSM-15-255-s001.pdf]

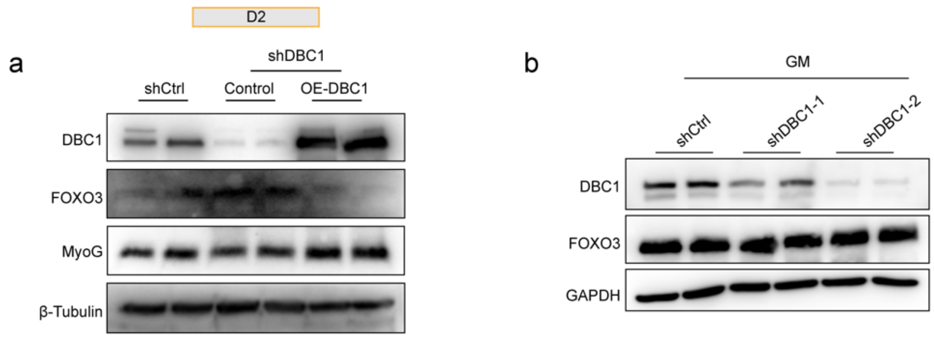

Supplementary Fig. 8 DBC1 negatively regulates FOXO3 during myogenesis

(a) Western blotting analysis for FOXO3 and MyoG protein levels in DBC1 knockdown C2C12 cells, DBC1 knockdown C2C12 cells that re-expressed DBC1 or the control cells that had been induced to differentiate for 2 days (D2). **(b)** Western blotting analysis for FOXO3 protein levels in proliferating DBC1 knockdown and the control C2C12 cells.
