# Supplementary material for: DBC1 maintains skeletal muscle integrity by enhancing myogenesis and preventing myofibre wasting
Source: J Cachexia Sarcopenia Muscle. 2023 Dec 7;15(1):255–69. doi: 10.1002/jcsm.13398 (PMC10834312; doi:10.1002/jcsm.13398)
Supplement: Supplementary file 9 — Figure S9. FOXO3 deletion in DBC1 knockdown C2C12 cells rescues myogenesis (a) Western blotting analysis for FOXO3 protein levels in DBC1 knockdown C2C12 cells that were added lentivirus to knock down FOXO3 for 48 h. (b and c) Western lotting analysis for MyoG protein levels (b) and MHC protein levels (c) in DBC1 knockdown C2C12 cells, DBC1 and FOXO3 double knockdown C2C12 cells and the control cells that had been induced to differentiate for 2 days and 7 days, respectively. [file JCSM-15-255-s004.pdf]

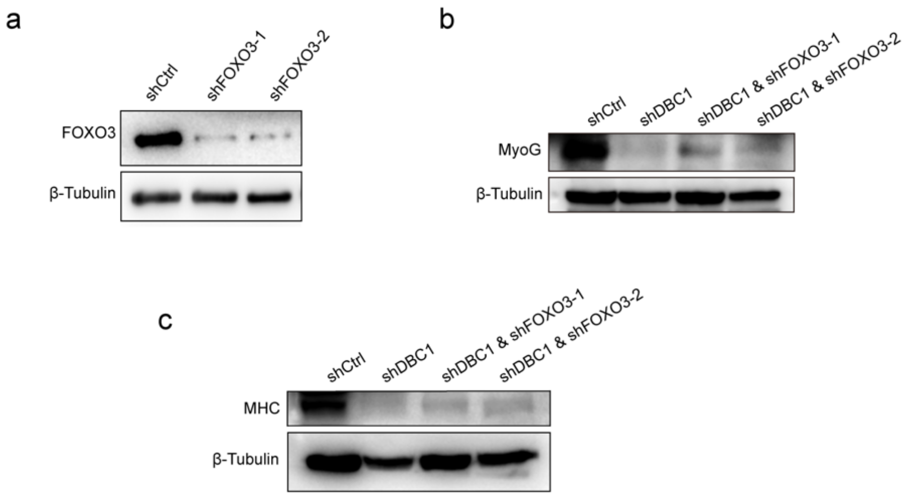

Supplementary Fig. 9 FOXO3 deletion in DBC1 knockdown C2C12 cells rescues myogenesis

(a) Western blotting analysis for FOXO3 protein levels in DBC1 knockdown C2C12 cells that were added lentivirus to knock down FOXO3 for 48 h. **(b and c)** Western blotting analysis for MyoG protein levels (b) and MHC protein levels (c) in DBC1 knockdown C2C12 cells, DBC1 and FOXO3 double knockdown C2C12 cells and the control cells that had been induced to differentiate for 2 days and 7 days, respectively.
