# Supplementary material for: DBC1 maintains skeletal muscle integrity by enhancing myogenesis and preventing myofibre wasting
Source: J Cachexia Sarcopenia Muscle. 2023 Dec 7;15(1):255–69. doi: 10.1002/jcsm.13398 (PMC10834312; doi:10.1002/jcsm.13398)
Supplement: Supplementary file 10 — Figure S10. FOXO3 expression is increased in old skeletal muscle (a) Immunofluorescence staining of FOXO3 (red) in TA muscles isolated from young (3 months) and old (24 months) C57BL / 6 J mice. Nuclei were counterstained with DAPI (blue). Scale bars = 100 μm. (b) Western blotting analysis for FOXO3 protein levels in TA muscles from young (3 months) and old (24 months) C57BL / 6 J mice. P values were calculated using two‐tailed Student's t‐test. [file JCSM-15-255-s014.pdf]

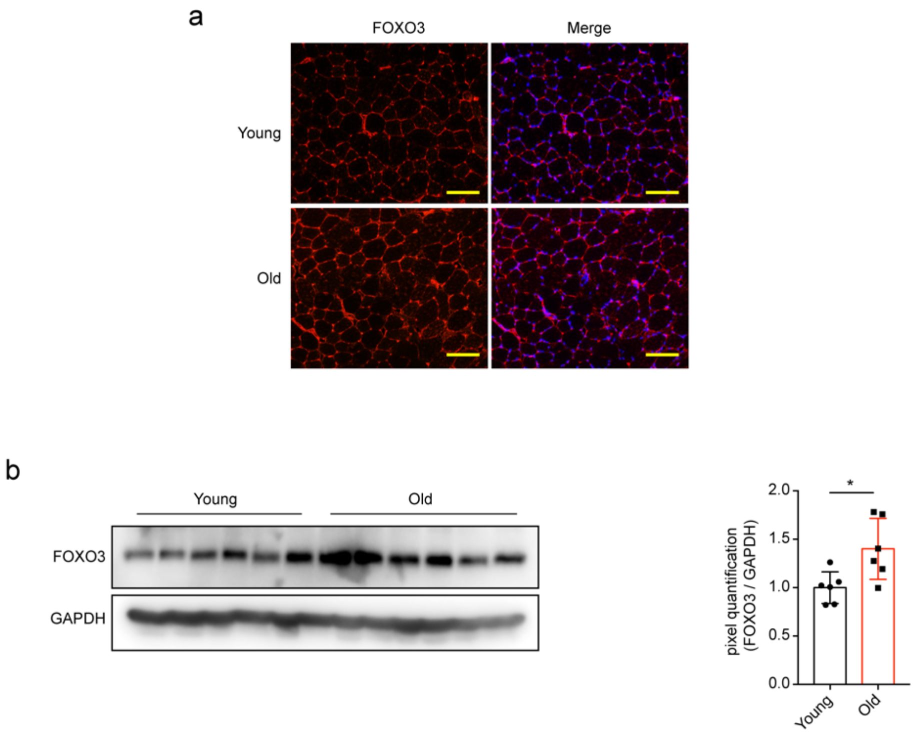

Supplementary Fig. 10 FOXO3 expression is increased in old skeletal muscle

(a) Immunofluorescence staining of FOXO3 (red) in TA muscles isolated from young (3 months) and old (24 months) C57BL / 6J mice. Nuclei were counterstained with DAPI (blue). Scale bars = 100 μm. **(b)** Western blotting analysis for FOXO3 protein levels in TA muscles from young (3 months) and old (24 months) C57BL / 6J mice. P values were calculated using two-tailed Student's t-test.
