# Supplementary material for: DBC1 maintains skeletal muscle integrity by enhancing myogenesis and preventing myofibre wasting
Source: J Cachexia Sarcopenia Muscle. 2023 Dec 7;15(1):255–69. doi: 10.1002/jcsm.13398 (PMC10834312; doi:10.1002/jcsm.13398)
Supplement: Supplementary file 11 — Figure S11. DBC1 knockdown does not affect mitochondrial functions in proliferating C2C12 cells (a) Mitochondrial membrane potential (MMP) of proliferating DBC1 knockdown and the control C2C12 cells that were analyzed by FACS. (b) The number of mitochondria of proliferating DBC1 knockdown and the control C2C12 cells were analyzed by FACS. (c) ATP production was accessed in vitro using cell lysates of proliferating DBC1 knockdown and the control C2C12 cells. (d) Measurements of ROS production of proliferating DBC1 knockdown and the control C2C12 cells, analyzed by FACS. P values were calculated using one‐way ANOVA for multiple comparison. [file JCSM-15-255-s011.pdf]

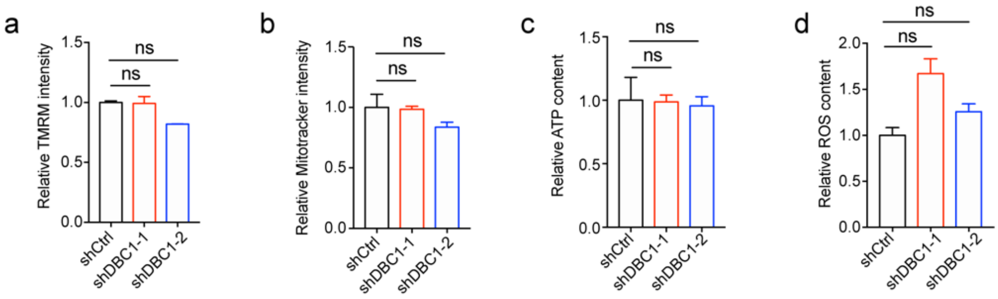

Supplementary Fig. 11 DBC1 knockdown does not affect mitochondrial functions in proliferating C2C12 cells

(a) Mitochondrial membrane potential (MMP) of proliferating DBC1 knockdown and the control C2C12 cells that were analyzed by FACS. **(b)** The number of mitochondria of proliferating DBC1 knockdown and the control C2C12 cells were analyzed by FACS. **(c)** ATP production was accessed in vitro using cell lysates of proliferating DBC1 knockdown and the control C2C12 cells. **(d)** Measurements of ROS production of proliferating DBC1 knockdown and the control C2C12 cells, analyzed by FACS. P values were calculated using one-way ANOVA for multiple comparison.
