# Supplementary material for: DBC1 maintains skeletal muscle integrity by enhancing myogenesis and preventing myofibre wasting
Source: J Cachexia Sarcopenia Muscle. 2023 Dec 7;15(1):255–69. doi: 10.1002/jcsm.13398 (PMC10834312; doi:10.1002/jcsm.13398)
Supplement: Supplementary file 12 — Figure S12. DBC1 negatively regulates the transcriptional activity and dephosphorylation of FOXO3 in differentiated cells (a) Relative gene expression of Atrogin1 and Murf1 in DBC1 knockdown myotubes, DBC1 knockdown myotubes treated by Carbenoxolone (CBX, 100 μM) and the control myotubes, determined by RT‐qPCR. (b‐c) Western blotting analysis for phosphorylated FOXO3 (p‐FOXO3) and total FOXO3 protein levels in DBC1 knockdown (b) and DBC1 overexpression (c) myotubes. The myotubes were fully differentiated for 7 days before infected for 2 days with lentiviruses that knocked down DBC1 or retroviruses that overexpressed DBC1. P values were calculated using one‐way ANOVA for multiple comparison. [file JCSM-15-255-s002.pdf]

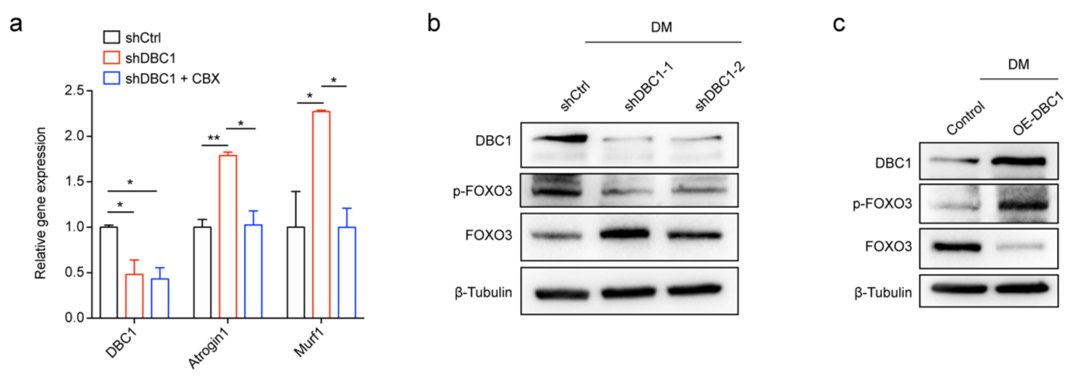

Supplementary Fig. 12 DBC1 negatively regulates the transcriptional activity and dephosphorylation of FOXO3 in differentiated cells

(a) Relative gene expression of *Atrogin1* and *Murf1* in DBC1 knockdown myotubes, DBC1 knockdown myotubes treated by Carbenoxolone (CBX, 100 μM) and the control myotubes, determined by RT-qPCR. **(b-c)** Western blotting analysis for phosphorylated FOXO3 (p-FOXO3) and total FOXO3 protein levels in DBC1 knockdown (b) and DBC1 overexpression (c) myotubes. The myotubes were fully differentiated for 7 days before infected for 2 days with lentiviruses that knocked down DBC1 or retroviruses that overexpressed DBC1. P values were calculated using one-way ANOVA for multiple comparison.
