# Supplementary material for: DBC1 maintains skeletal muscle integrity by enhancing myogenesis and preventing myofibre wasting
Source: J Cachexia Sarcopenia Muscle. 2023 Dec 7;15(1):255–69. doi: 10.1002/jcsm.13398 (PMC10834312; doi:10.1002/jcsm.13398)
Supplement: Supplementary file 13 — Figure S13. DBC1 negatively regulates the phosphorylation of FOXO3 in proliferating C2C12 cells (a) Western blotting analysis for phosphorylated FOXO3 (p‐FOXO3) and total FOXO3 protein levels in DBC1 knockdown and the control proliferating C2C12 cells. (b) (Left) Representative images of Immunofluorescence staining of FOXO3 (red) in DBC1 knockdown and the control proliferating C2C12 cells. Nuclei were counterstained with DAPI (blue). Scale bars = 400 μm. (Right) Quantification of the nuclear / cytoplasmic fluorescence ratio. P values were calculated using oneway ANOVA for multiple comparison. [file JCSM-15-255-s016.pdf]

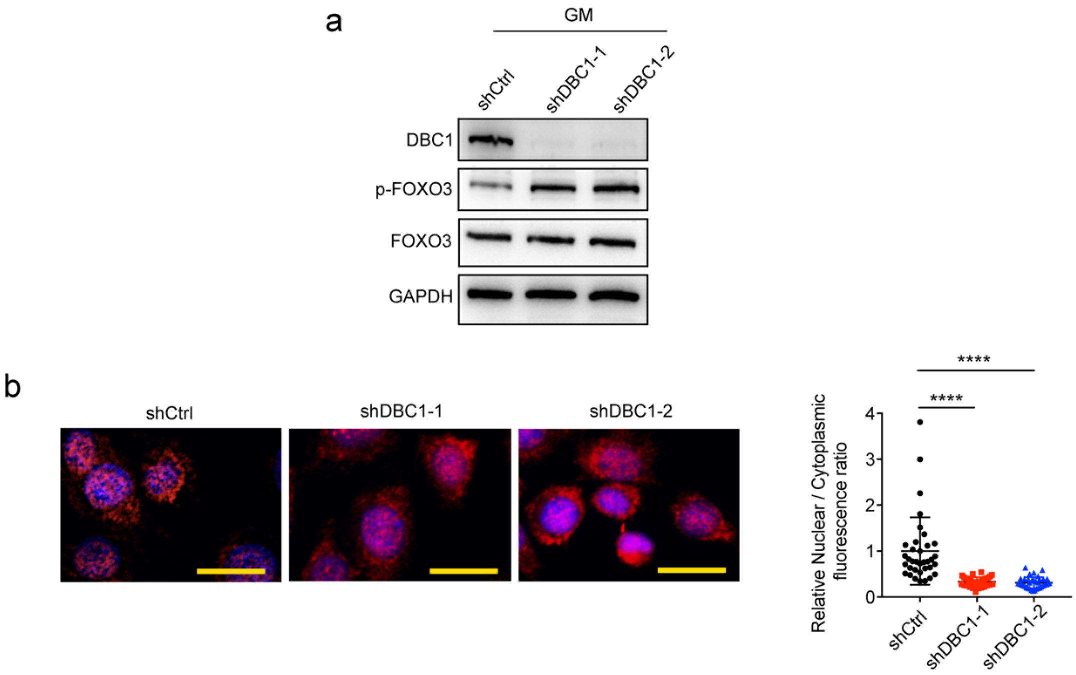

Supplementary Fig. 13 DBC1 negatively regulates the phosphorylation of FOXO3 in proliferating C2C12 cells

(a) Western blotting analysis for phosphorylated FOXO3 (p-FOXO3) and total FOXO3 protein levels in DBC1 knockdown and the control proliferating C2C12 cells. (b) (Left) Representative images of Immunofluorescence staining of FOXO3 (red) in DBC1 knockdown and the control proliferating C2C12 cells. Nuclei were counterstained with DAPI (blue). Scale bars = 400 µm. (Right) Quantification of the nuclear / cytoplasmic fluorescence ratio. P values were calculated using one-way ANOVA for multiple comparison.
