# Supplementary material for: DBC1 maintains skeletal muscle integrity by enhancing myogenesis and preventing myofibre wasting
Source: J Cachexia Sarcopenia Muscle. 2023 Dec 7;15(1):255–69. doi: 10.1002/jcsm.13398 (PMC10834312; doi:10.1002/jcsm.13398)
Supplement: Supplementary file 14 — Figure S14. DBC1 positively regulates MDM2 expression (a) Relative gene expression of MDM2 in proliferating DBC1 knockdown and the control C2C12 cells, determined by RT‐qPCR. (b) Western blotting analysis for MDM2 protein levels in proliferating DBC1 knockdown C2C12 cells and the control cells. P values were calculated using two‐tailed Student's t‐test. [file JCSM-15-255-s006.pdf]

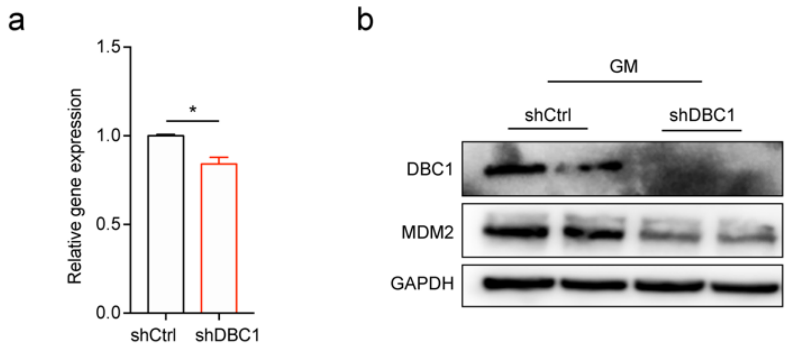

Supplementary Fig. 13 DBC1 positively regulates MDM2 expression

(a) Relative gene expression of *MDM2* in proliferating DBC1 knockdown and the control C2C12 cells, determined by RT-qPCR. **(b)** Western blotting analysis for MDM2 protein levels in proliferating DBC1 knockdown C2C12 cells and the control cells. P values were calculated using two-tailed Student's t-test.
