# Supplementary material for: DBC1 maintains skeletal muscle integrity by enhancing myogenesis and preventing myofibre wasting
Source: J Cachexia Sarcopenia Muscle. 2023 Dec 7;15(1):255–69. doi: 10.1002/jcsm.13398 (PMC10834312; doi:10.1002/jcsm.13398)
Supplement: Supplementary file 15 — Figure S15. Ubiquitinated FOXO3 is degraded via proteasome pathway (a and b) Western blotting analysis for FOXO3 and MyoG protein levels in C2C12 cells that had been induced to differentiate for 0 h, 3 h or 6 h with the treatment of MG‐ 132 (1 μM) (a) or Leupeptin (10 μM) (b). [file JCSM-15-255-s009.pdf]

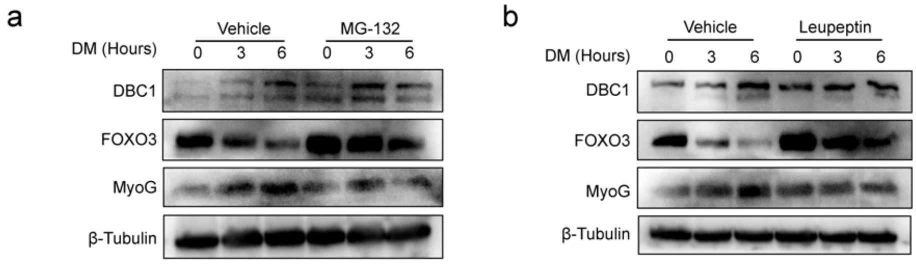

Supplementary Fig. 14 Ubiquitinated FOXO3 is degraded via proteasome pathway

(a and b) Western blotting analysis for FOXO3 and MyoG protein levels in C2C12 cells that had been induced to differentiate for 0 h, 3 h or 6 h with the treatment of MG-132 (1 μM) (a) or Leupeptin (10 μM) (b).
