# Supplementary material for: DBC1 maintains skeletal muscle integrity by enhancing myogenesis and preventing myofibre wasting
Source: J Cachexia Sarcopenia Muscle. 2023 Dec 7;15(1):255–69. doi: 10.1002/jcsm.13398 (PMC10834312; doi:10.1002/jcsm.13398)
Supplement: Supplementary file 16 — Data S1. Supporting Information [file JCSM-15-255-s005.docx]

SUPPLEMENTARY INFORMATION

DBC1 maintains skeletal muscle integrity by enhancing myogenesis and preventing myofiber wasting

Na Liang^1^, Jia He^1^, Jiaqi Yan^1^, Xueying Han^1^, Xiaoqian Zhang^1^, **Yamei Niu^2^**, Wuga sha^1^, Jun Li^1*^

*Correspondence: [Jun_Li@ibms.pumc.edu.cn](mailto:Jun_Li@ibms.pumc.edu.cn)

**Supplementary Methods:**

**RNA sequencing**

Total RNA was isolated using Trizol reagent (Invitrogen). RNA libraries and transcriptome sequencing were performed by Novogene (China). Differentially expressed genes were filtered using a change greater than 1.5-fold and p-value < = 0.05. Gene ontology biological process (GO) and The Kyoto Encyclopedia of Genes and Genomes (KEGG) pathway were analyzed using The Database for Annotation, Visualization and Integrated Discovery (DAVID; https://david.abcc.ncifcrf.gov/).

**Measurement of ATP**

ATP content was detected using ATP Determination kit (Invitrogen, A22066) according to the instructions. Briefly, **Cells were lysed in** Triton X-100 lysis buffer and the protein concentrations were determined by the Bradford protein assay (Solarbio, **PC0010**). **Proteins in the lysates were** removed using 10 kD ultrafiltration tubes. **Luminescence signal were measured on using the BioTek Synergy H1 Synergy H1 microplate reader (Agilent).** ATP content was calculated by **normalization** to protein concentration.

**Immunoprecipitation**

Protein were extracted in **Triton X-100** lysis buffer (150 mM NaCl, 1 mM EDTA, 1 mM EGTA, 1 % Triton X-100, 0.5% NP-40, 10 mM Tris HCl, pH 7.4) added with protease inhibitor cocktail (MCE, HY-K0010). Protein concentrations were determined by the Bradford protein assay. The lysates were incubated with Protein A/G agarose beads (Millipore, IP05) and the corresponding antibodies overnight at 4^o^C. The beads were washed 3 times using Triton X-100 lysis buffer, followed by elution. Then proteins were **applied to** SDS-PAGE **and western blotting test** with proper antibodies.

**Apoptosis assay**

Cell apoptosis was assessed using Annexin V / PI kit (BD Biosciences, 556547). **Cells were stained by FITC Annexin V and PI (BD, 556547) in PBS buffer for 15 min at room temperature, followed by measurement** on BD AccuriC6 (Becton, Dickinson and Company). Data were analyzed using FlowjoVX10.

**Virus production and infection**

For lentivirus production, plasmids of empty vector pLKO.1 (Addgene plasmid # 8453), **pLKO.1-shDBC1 (designed and purchased from Ribo Bio),** packaging plasmids psPAX2 (Addgene plasmid #12260) and pMD2.G (Addgene plasmid #12259) were transfected into 293T cells using Lipofectamine 2000 (Thermo, 11668019), according to the manufacturer’s instructions. The virus-containing media was harvested 48 h post-transfection and filtered through 0.45 μm filters. The C2C12 cells were infected by filtered media in the presence of 8 μg/mL polybrene for 48 h (Sigma, H9268). After infection, stably transfected DBC1 knockdown C2C12 cells were selected with 2 μg/mL puromycin.

DBC1 overexpression C2C12 cells were generated using retrovirus. Retrovirus expressed empty vector pMSCV **(Addgene plasmid #111630)**, DBC1 overexpression vector pMSCV-DBC1 **(made by our group)** and packaging plasmids Gag-pol (Addgene plasmid #14887) and VSV-G (Addgene plasmid #8454) were transfected into 293T cells using Lipofectamine 2000 (Thermo, 11668019). The remaining procedures were similar to those for generating DBC1 knockdown C2C12 cells.

**RT-PCR**

Total RNA was extracted using the Trizol reagent (Invitrogen, 15596026) and the cDNA synthesis was performed with iScript TM cDNA Synthesis Kit (Bio-Rad, 1708891). RT-PCR was performed using NEBNext® Q5® Hot Start HiFi PCR Master Mix (NEB, M0543S) on a LightCycler® 384 Real-Time PCR System (Roche). Gene expression was normalized to housekeeping gene PPIA. The primers for RT-qPCR are listed below:

Mouse PPIA-F: 5’-GAGCTGTTTGCAGACAAAGTTC-3’,

Mouse PPIA-R: 5’-CCCTGGCACATGAATCCTGG-3’,

Mouse DBC1-F: 5’-AACAGATTTCCTGCTCGGGG-3’,

Mouse DBC1-R: 5’-CTTAGCACCCCAAGGCTCTC-3’,

Mouse MyoG-F: 5’-AGCGCAGGCTCAAGAAAGTGAATG-3’,

Mouse MyoG-R: 5’-CTGTAGGCGCTCAATGTACTGGAT-3’

Mouse MHC-F: 5’-CTTGGTGGACAAACTACAGACT-3’,

Mouse MHC-R: 5’-TGCAGAATTTATTTCCGTGAT-3’,

Mouse HK2-F: 5’- AACCTCAAAGTGACGGTGGG-3’,

Mouse HK2-R: 5’- TCACATTTCGGAGCCAGATCT-3’,

Mouse LDHA-F: 5’- TCAACCTGGTCCAGCGAAAC-3’,

Mouse LDHA-R: 5’- CCACTGGATTGGAGACGATCA-3’,

Mouse Pfkm-F: 5’- CAGATCAGTGCCAACATAACCAA-3’,

Mouse Pfkm-R: 5’- CGGGATGCAGAGCTCATCA-3’,

Mouse Cs-F: 5’- TTGTACAGCTGAGCCACCAG-3’,

Mouse Cs-R: 5’- TGGTCCCAGGATACGGTCAT-3’,

Mouse SIRT1-F: 5’- TGACAGAACGTCACACGCC-3’

Mouse SIRT1-R: 5’- AACAATCTGCCACAGCGTCA-3’,

Mouse FOXO3-F: 5’- CTGGGGGAACCTGTCCTATG-3’,

Mouse FOXO3-R: 5’- TCATTCTGAACGCGCATGAAG-3’,

Mouse MDM2-F: 5’- ATGAGGTCTATCGGGTCACAG-3’,

Mouse MDM2-R: 5’- CACATCCAAGCCTTCTTCTGC-3’

**Proteasome activity**

Proteasome activity in total homogenates from myotubes were determined by evaluating the cleavage of specific fluorogenic substrates as formerly described (S22). Specifically, myotubes were homogenized in lysis buffer (50 mM Tris HCl pH 7.5, 250 mM Sucrose, 5 mM MgCl_2_, 0.5 mM EDTA, 2 mM ATP, and 1 mM DTT). The supernatant was collected **after centrifuging** and protein concentration was determined using the Bradford protein assay. 20 μg proteins **were mixed with** 100 μM of the fluorogenic substrate succinyl-Leu-Leu-Val-Tyr-7-amino-4-methylcoumarin (MCE, 94367-21-2), **followed by incubation** at 37 °C for 60 min. Proteasomal inhibitor MG-132 (20 μM, MCE, S2619) was added as negative control. Fluorescence was read on a **FlexStation 3 Multi-Mode Microplate Reader (Molecular Devices, America)** at wavelength of 390 nm (excitation) and 460 nm (emission). The activity was expressed as units of fluorescence per microgram of protein.

**Supplementary references:**

S1. Sartori R, Romanello V, Sandri M. Mechanisms of muscle atrophy and hypertrophy: implications in health and disease. Nat Commun. 2021;12(1):330.

S2. Yang J, Cao RY, Li Q, Zhu F. Muscle Atrophy in Cancer. Adv Exp Med Biol. 2018;1088:329-46.

S3. Fong AP, Tapscott SJ. Skeletal muscle programming and re-programming. Curr Opin Genet Dev. 2013;23(5):568-73.

S4. Relaix F, Bencze M, Borok MJ, Der Vartanian A, Gattazzo F, Mademtzoglou D, et al. Perspectives on skeletal muscle stem cells. Nat Commun. 2021;12(1):692.

S5. Gomes MJ, Martinez PF, Pagan LU, Damatto RL, Cezar MDM, Lima ARR, et al. Skeletal muscle aging: influence of oxidative stress and physical exercise. Oncotarget. 2017;8(12):20428-40.

S6. Ludikhuize MC, Rodríguez Colman MJ. Metabolic Regulation of Stem Cells and Differentiation: A Forkhead Box O Transcription Factor Perspective. Antioxid Redox Signal. 2021;34(13):1004-24.

S7. Milan G, Romanello V, Pescatore F, Armani A, Paik JH, Frasson L, et al. Regulation of autophagy and the ubiquitin-proteasome system by the FoxO transcriptional network during muscle atrophy. Nat Commun. 2015;6:6670.

S8. Fang Q, Bellanti JA, Zheng SG. Advances on the role of the deleted in breast cancer (DBC1) in cancer and autoimmune diseases. J Leukoc Biol. 2021;109(2):449-54.

S9. Ryall JG, Dell'Orso S, Derfoul A, Juan A, Zare H, Feng X, et al. The NAD(+)-dependent SIRT1 deacetylase translates a metabolic switch into regulatory epigenetics in skeletal muscle stem cells. Cell Stem Cell. 2015;16(2):171-83.

S10. Gomes MD, Lecker SH, Jagoe RT, Navon A, Goldberg AL. Atrogin-1, a muscle-specific F-box protein highly expressed during muscle atrophy. Proc Natl Acad Sci U S A. 2001;98(25):14440-5.

S11. Lee JH, Park JW, Kang KS, Park TS. Forkhead box O3 promotes cell proliferation and inhibits myotube differentiation in chicken myoblast cells. Br Poult Sci. 2019;60(1):23-30.

S12. Che J, Xu C, Wu Y, Jia P, Han Q, Ma Y, et al. MiR-1290 promotes myoblast differentiation and protects against myotube atrophy via Akt/p70/FoxO3 pathway regulation. Skelet Muscle. 2021;11(1):6.

S13. Folmes CD, Dzeja PP, Nelson TJ, Terzic A. Metabolic plasticity in stem cell homeostasis and differentiation. Cell Stem Cell. 2012;11(5):596-606.

S14. Yucel N, Wang YX, Mai T, Porpiglia E, Lund PJ, Markov G, et al. Glucose Metabolism Drives Histone Acetylation Landscape Transitions that Dictate Muscle Stem Cell Function. Cell Rep. 2019;27(13):3939-55 e6.

S15. Marco Sandri, Claudia Sandri, Alex Gilbert, Carsten Skurk, Elisa Calabria, Anne Picard, et al. Foxo Transcription Factors Induce the Atrophy-Related Ubiquitin Ligase Atrogin-1 and Cause Skeletal Muscle Atrophy. Cell. 2004;117(3):399-412.

S16. Fu W, Ma Q, Chen L, Li P, Zhang M, Ramamoorthy S, et al. MDM2 acts downstream of p53 as an E3 ligase to promote FOXO ubiquitination and degradation. J Biol Chem. 2009;284(21):13987-4000.

S17. Moreno-Navarrete JM, Moreno M, Vidal M, Ortega F, Serrano M, Xifra G, et al. Deleted in breast cancer 1 plays a functional role in adipocyte differentiation. Am J Physiol Endocrinol Metab. 2015;308(7):E554-61.

S18. Hori S, Hiramuki Y, Nishimura D, Sato F, Sehara-Fujisawa A. PDH-mediated metabolic flow is critical for skeletal muscle stem cell differentiation and myotube formation during regeneration in mice. Faseb j. 2019;33(7):8094-109.

S19. Pala F, Di Girolamo D, Mella S, Yennek S, Chatre L, Ricchetti M, et al. Distinct metabolic states govern skeletal muscle stem cell fates during prenatal and postnatal myogenesis. J Cell Sci. 2018;131(14).

S20. Gouspillou G, Bourdel-Marchasson I, Rouland R, Calmettes G, Biran M, Deschodt-Arsac V, et al. Mitochondrial energetics is impaired in vivo in aged skeletal muscle. Aging Cell. 2014;13(1):39-48.

S21. Greer EL, Oskoui PR, Banko MR, Maniar JM, Gygi MP, Gygi SP, et al. The energy sensor AMP-activated protein kinase directly regulates the mammalian FOXO3 transcription factor. J Biol Chem. 2007;282(41):30107-19.

S22. Jessica Segalés, Eusebio Perdiguero, Antonio L. Serrano, Pedro Sousa-Victor,

Laura Ortet, Mercè Jardí, et al. Sestrin prevents atrophy of disused and aging muscles

by integrating anabolic and catabolic signals. Nat Commun. 2020;11:189.
